# Pandemic influenza virus vaccines boost hemagglutinin stalk-specific antibody responses in primed adult and pediatric cohorts

**DOI:** 10.1038/s41541-019-0147-z

**Published:** 2019-12-06

**Authors:** Raffael Nachbagauer, Bruno Salaun, Daniel Stadlbauer, Mohammad A. Behzadi, Damien Friel, Arvind Rajabhathor, Angela Choi, Randy A. Albrecht, Muriel Debois, Adolfo García-Sastre, Ronan N. Rouxel, Weina Sun, Peter Palese, Corey P. Mallett, Bruce L. Innis, Florian Krammer, Carine Claeys

**Affiliations:** 1 Department of Microbiology, Icahn School of Medicine at Mount Sinai, New York, NY USA; 2 GSK, Rixensart, Belgium; 3 GSK, Wavre, Belgium; 4 Graduate School of Biomedical Services, Icahn School of Medicine at Mount Sinai, New York, NY USA; 5 Global Health and Emerging Pathogens Institute, Icahn School of Medicine at Mount Sinai, New York, NY USA; 6 Department of Medicine, Icahn School of Medicine at Mount Sinai, New York, NY USA; 7 GSK, Rockville, MD 20850 USA; 8 GSK, King of Prussia, PA USA; 9 GSK, Rixensart, Belgium; 10 Present Address: PATH, Washington, DC, USA; 11 Present Address: Spmt-Arista Asbl, Brussel, Belgium

**Keywords:** Antibodies, Influenza virus, Inactivated vaccines, Adjuvants, Immunological memory

## Abstract

Licensed influenza virus vaccines target the head domain of the hemagglutinin (HA) glycoprotein which undergoes constant antigenic drift. The highly conserved HA stalk domain is an attractive target to increase immunologic breadth required for universal influenza virus vaccines. We tested the hypothesis that immunization with a pandemic influenza virus vaccine boosts pre-existing anti-stalk antibodies. We used chimeric cH6/1, full length H2 and H18 HA antigens in an ELISA to measure anti-stalk antibodies in recipients participating in clinical trials of A/H1N1, A/H5N1 and A/H9N2 vaccines. The vaccines induced high titers of anti-H1 stalk antibodies in adults and children, with higher titers elicited by AS03-adjuvanted vaccines. We also observed cross-reactivity to H2 and H18 HAs. The A/H9N2 vaccine elicited plasmablast and memory B-cell responses. Post-vaccination serum from vaccinees protected mice against lethal challenge with cH6/1N5 and cH5/3N4 viruses. These findings support the concept of a chimeric HA stalk-based universal influenza virus vaccine. clinicaltrials.gov: NCT02415842.

## Influenza vaccine: boosting immunity to hemagglutinin stalk domains

The head domain of influenza virus hemagglutinin (HA), the main target of licensed influenza virus vaccines, undergoes constant antigenic drift. The HA stalk domain, on the other hand, is highly conserved and is thus an attractive target for developing universal influenza vaccine formulations. Raffael Nachbagauer and colleagues now show that vaccination with pandemic influenza virus vaccines boosts pre-existing antibody responses to HA stalk domains in pediatric cohorts. Analysis of serum from individuals immunized with pandemic vaccines A/H1N1, A/H5N1 and A/H9N2, revealed basal levels of anti-stalk antibodies that were increased following immunization. The elicited antibodies had neutralization properties, and plasmablast responses from peripheral blood immune cells recovered from vaccinated individuals were also recorded. These findings support pandemic vaccines as a potential strategy towards universal influenza virus vaccines by expanding pre-existing antibodies against conserved HA stalk structures.

## Introduction

Influenza is a major public health burden with substantial clinical and economic impact.^1^ Vaccination is the cornerstone of influenza prevention, and is recommended by the World Health Organization (WHO) for young children, the elderly, pregnant women, people with certain chronic medical conditions, and health care workers.^2^

Hemagglutinin (HA) and neuraminidase (NA) are the two major glycoproteins on the influenza virus surface. HA consists of a membrane-distal globular head domain and a membrane-proximal stalk domain. During influenza virus infection, the virus enters the host cell via binding of the virus HA globular head to sialylated receptors of the host cell.^3^ Current influenza virus vaccines act by inducing serum neutralizing antibodies that target the HA head domain.^4^ The head domain is immunodominant, and most of the humoral immune response elicited by influenza virus vaccines is directed towards it.^5^ It has high plasticity and undergoes constant antigenic drift,^6–8^ meaning that seasonal influenza virus vaccines must be reformulated almost every year to allow them to induce antibodies that recognize the new variants produced.^9^

The WHO makes annual recommendations on seasonal influenza virus vaccine composition based on a prediction of which viral strains will predominate in the upcoming season.^9^ In some seasons, the predictions are imperfect, resulting in mismatch of the viral vaccine strains with the circulating strains and associated poor vaccine effectiveness.^10^ Moreover, manufacture of a new vaccine formulation using current egg-based production followed by inactivation and detergent splitting of the vaccine virus takes several months which is clearly suboptimal. A game-changing universal influenza virus vaccine that would elicit durable immunity to all influenza virus strains and subtypes could overcome these issues and is an area of focus for the vaccine industry.

Influenza virus type A HAs are phylogenetically classified into group 1 and group 2 HAs. In contrast to the HA head domain, the stalk domain is relatively conserved within these respective groups. Anti-stalk antibodies are known to protect against a wide range of influenza virus strains and subtypes in animal models, broadly following this phylogeny.^11,12^ The stalk domain therefore represents an attractive target for the development of a universal influenza virus vaccine. Vaccination with constructs comprising the stalk domain but lacking the head domain (a mini HA construct) has been shown to induce an immune response in preclinical studies,^13–15^ and a Phase I clinical trial is underway using this approach (NCT03814720). An alternative approach is to employ sequential immunization with a series of chimeric influenza viruses with divergent HA head domains and conserved stalk domains to overcome the immunodominance of the head domain and redirect the immune system towards the stalk domain.^16,17^ Use of an adjuvant has been shown to increase the magnitude of the humoral immune response.^18–20^

Adult humans have pre-existing immunity to the HA stalk domain from previous seasonal influenza virus exposure.^21^ Immunization with an influenza virus vaccine containing an HA from an influenza virus strain not circulating in humans should theoretically boost these pre-existing anti-stalk antibodies since the head domain will not be recognized and memory B-cells with specificities in the conserved stalk domain will be recalled.^10,11^ Thus, a vaccine containing pandemic or pre-pandemic influenza viruses can act as a surrogate for one dose of a chimeric HA-based broadly reactive vaccine. This effect has been observed in previous studies that measured levels of HA stalk antibodies after H5 and H7 influenza virus vaccination.^22–24^ In the present study, we further tested this hypothesis using assays that measure HA stalk antibodies in serum obtained from previous clinical trials with different vaccines containing pandemic or pre-pandemic viruses (i.e., A/H1N1, A/H5N1, A/H9N2) or with seasonal influenza virus vaccine.

## Results

The clinical trials included^25–32^ and the number of participants is shown in Table 1. Demographic characteristics and pre-vaccination seropositivity for HI against the vaccine homologous virus and the A/H1N1pdm09 virus are shown in Supplementary Tables 1 and 2, respectively.

**Table 1:** Clinical trials, participants, and vaccines.

Trial	Trial group	*N*	Vaccination schedule	Immune response sampling schedule
*Homologous prime-boost studies in adults*				
Trial 1: A/H1N1^25^	A/H1N1 AS03	29	A/California/07/2009 3.75 µg + AS03_A_ on D0 and D21 Seasonal IIV3 on D42	D0, D21, D42, D182
	A/H1N1 non-adjuvanted	29	A/California/07/2009 15 µg on D0 and D21 Seasonal IIV3 on D42	
Trial 2: A/H5N1^26^	A/H5N1 AS03	29	A/Indonesia/5/2005 3.75 µg + AS03_A_ on D0 and D21	D0, D21, D42, D182, D385
	A/H5N1 non-adjuvanted	27	A/Indonesia/5/2005 15 µg on D0 and D21	
Trial 3: A/H9N2^27^	A/H9N2 AS03	30	A/chicken/Hong Kong/G9/1997 3.75 µg + AS03_A_ on D0 and D21	D0, D21, D42, D182
	A/H9N2 non-adjuvanted	30	A/chicken/Hong Kong/G9/1997 15 µg on D0 and D21	
*Seasonal IIV4 study in adults*				
Trial 4: IIV4^28^	IIV4 non-adjuvanted	30	Seasonal IIV4 15 µg per strain at D0 A/Christchurch/16/2010 (H1N1 pdm09) A/Texas/50/2012 (H3N2) B/Massachusetts/02/2012 (B Yamagata) B/Brisbane/60/2008 (B Victoria)	D0, D21
*Heterologous prime-boost studies in adults*				
Trial 5: A/H5N1 booster^29^	A/H5N1 AS03 Indonesia > Turkey	26	A/Indonesia/5/05 3.75 µg + AS03_A_ on D0 Placebo on D182 A/turkey/Turkey/1/2005 3.75 µg + AS03_A_ on D549	D0, D42, D182, D224, D549, D591, D729
	A/H5N1 AS03 Turkey > Turkey	29	Placebo on D0 A/turkey/Turkey/1/2005 3.75 µg + AS03_A_ on D182 and D549	
Trial 6: A/H5N1 booster^30,31^	A/H5N1 AS03 Vietnam > Vietnam	53	A/Vietnam/1194/2004 3.75 µg + AS03_A_ on D0 and D365	D0, D21, D182, D365, D385, D549
	A/H5N1 AS03 Vietnam > Indonesia	49	A/Vietnam/1194/2004 + AS03_A_ on D0 A/Indonesia/5/05 3.75 µg + AS03_A_ on D365	
*Homologous prime-boost study in children*				
Trial 7: A/H5N1^32^	A/H5N1 AS03	33	A/Indonesia/5/2005 1.90 µg + AS03_B_ at D0 and D21	D0, D21, D42, D385
	Placebo	20	Placebo at D0 and D21	

^a^Trials 1, 2 and 3: serum samples from participants who received adjuvanted vaccine were randomly selected, and samples matched by age and study center were then selected from the non-adjuvanted group

^b^Trial 4: serum samples from participants 18−39 years of age were randomly selected

^c^Trials 5 and 6: all evaluable samples from eligible participants were used (i.e. no random selection)

^d^Trial 7: only samples from children (6−35 months) who were seronegative for A/H1N1pdm09 (A/California/07/2009-like) antibodies were selected to ensure that the children had not been primed through prior exposure to A/H1N1 virus. Not all study groups in the original trials were included; only the vaccines that were administered to participants whose samples were used in the present study are shown. IIV3: trivalent inactivated influenza vaccine; IIV4: quadrivalent inactivated influenza vaccine

### ELISA

#### Anti-H1 stalk antibodies

**Fig. 1:**
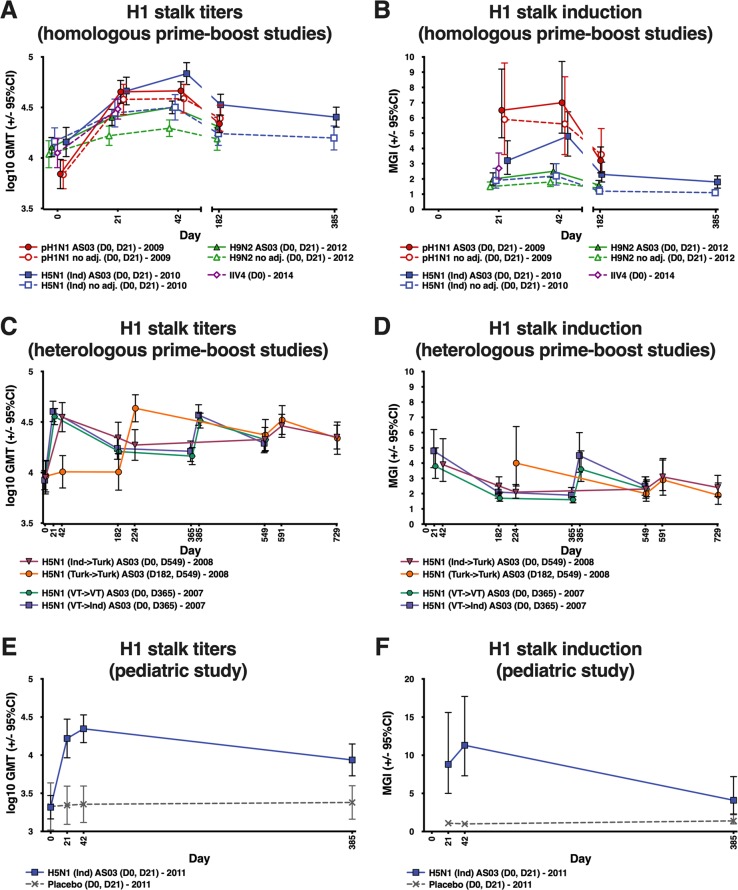
GMTs and MGIs for anti-H1 stalk antibodies measured by ELISA following vaccination with adjuvanted and non-adjuvanted vaccines. GMT and MGI values with 95% CI and group sizes are shown in Supplementary Table 3. Ind: A/Indonesia/5/05; Turk: A/turkey/Turkey/1/2005; VT: A/Vietnam/1194/2004. Error bars indicate 95% confidence intervals. CI: confidence interval; ELISA: enzyme-linked immunosorbent assay; GMT: geometric mean titer; IIV4: inactivated quadrivalent influenza vaccine; MGI: mean geometric increase.

All adult participants were ELISA-positive (≥66 EU/mL) for anti-H1 stalk antibodies pre-vaccination. Pre-vaccination GMTs were approximately 7000 EU/mL in the A/H1N1 study (trial 1), whilst pre-vaccination GMTs ranged from 8500 to 14,000 EU/mL in the other adult studies. In the homologous prime-boost adult studies (trials 1−3), vaccination with pandemic A/H1N1, A/H5N1 and A/H9N2 vaccines elicited an immune response against the H1 stalk (Fig. 1a). GMTs rose substantially after the first vaccine dose, followed by a very limited rise or no rise following the second dose administered 21 days later (Fig. 1a; Supplementary Table 3). GMTs remained higher than at baseline 6 months after vaccination (and at 12 months for the A/H5N1 vaccine), and declined at a similar rate with each vaccine (Fig. 1a). The seasonal IIV4 (trial 4) also elicited anti-H1 stalk antibodies (Fig. 1a). Most (90%) participants in this trial were seropositive for HI at baseline to the A/H1N1 vaccine-homologous virus (A/Christchurch/16/2010; Supplementary Table 2). MGIs were higher with the A/H1N1 vaccine than with the A/H5N1 or A/H9N2 vaccines (Fig. 1b; Supplementary Table 3).

An adjuvant effect was observed for all pandemic vaccines, although the A/H1N1 vaccine elicited a strong H1 stalk response with or without an adjuvant. The GMT ratios (adjuvanted/non-adjuvanted) 21 days after the first vaccine dose were 1.12 (95% CI: 0.73, 1.72) for the A/H1N1 vaccine, 1.66 (1.12, 2.47) for the A/H5N1 vaccine, and 1.39 (1.14, 1.69) for the A/H9N2 vaccine. Corresponding values for the difference in the percentage of participants with ≥4-fold rise in titer (adjuvanted minus non-adjuvanted) were 10.8 (−16.5, 36.8), 27.1 (6.0, 47.0), and 3.3 (−13.1, 20.2).

The pattern of the anti-H1 stalk response in the heterologous prime-boost studies (trials 5 and 6) reflected that of trials 1−3 (Fig. 1c, d; Supplementary Table 3). The stalk response with A/Indonesia/5/2005 (A/H5N1 Indonesia) followed by A/turkey/Turkey/1/2005 (A/H5N1 Turkey) administered 18 months later was similar to the response with two doses of A/H5N1 Turkey administered 12 months apart (trial 5). Similar findings were observed in trial 6 (Fig. 1c, d; Supplementary Table 3). Both booster vaccinations elicited similarly high titers as the primary vaccination.

All children were ELISA-positive for anti-H1 stalk antibodies pre-vaccination, with GMTs of approximately 2000 EU/mL (trial 7). Post-vaccination anti-H1 stalk antibody GMTs increased in the AS03-adjuvanted A/H5N1 vaccine group but titers were lower than in adults (Fig. 1e, f; Supplementary Table 3). There was no increase in GMTs in the placebo group.

#### Anti-H2 and anti-H18 stalk antibodies

**Fig. 2:**
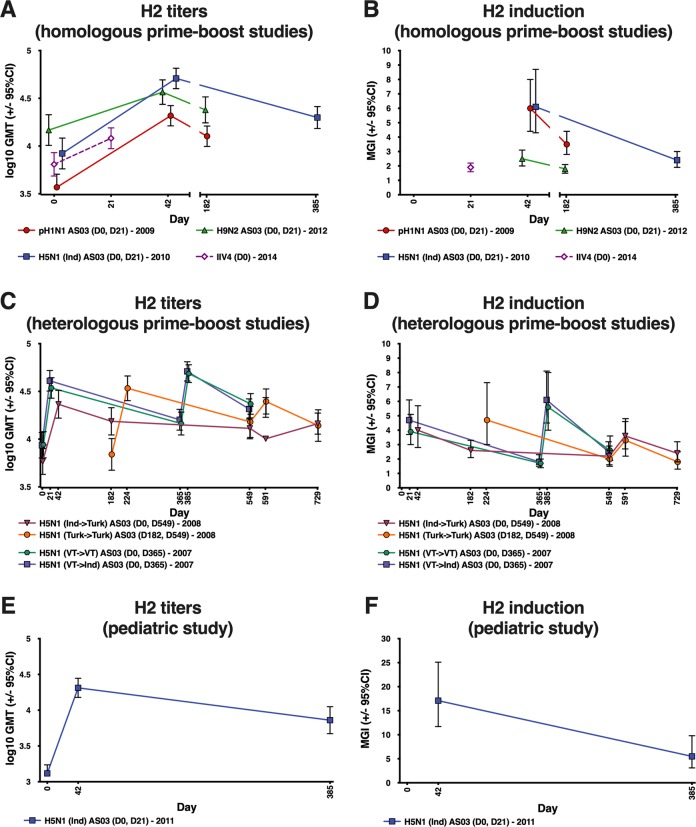
GMTs and MGIs for anti-H2 full-length antibodies measured by ELISA following vaccination with adjuvanted pandemic vaccines and seasonal vaccine. GMT and MGI values with 95% CI and group sizes are shown in Supplementary Table 4. Ind: A/Indonesia/5/05; Turk: A/turkey/Turkey/1/2005; VT: A/Vietnam/1194/2004. Error bars indicate 95% confidence intervals. CI: confidence interval; ELISA: enzyme-linked immunosorbent assay; GMT: geometric mean titer; IIV4: inactivated quadrivalent influenza vaccine; MGI: mean geometric increase.

Response was evaluated at 21 days after the second vaccine dose because the highest antibody titers were expected at this timepoint. Similar patterns as for the H1 stalk response were observed for H2 and H18 antibodies (Fig. 2a–f; Supplementary Table 4; Supplementary Table 5; Supplementary Fig. 1). Antibody levels following administration of the IIV4 were lower compared with the adjuvanted pandemic vaccinations (Fig. 2a). In the heterologous prime-boost studies (trials 5 and 6), antibody responses were similar with the regimens using two doses of A/H5N1 Turkey or Vietnam compared with the regimens using A/H5N1 Indonesia followed by A/Turkey or A/Vietnam followed by A/Indonesia (Fig. 2c).

### Microneutralization assay

**Fig. 3:**
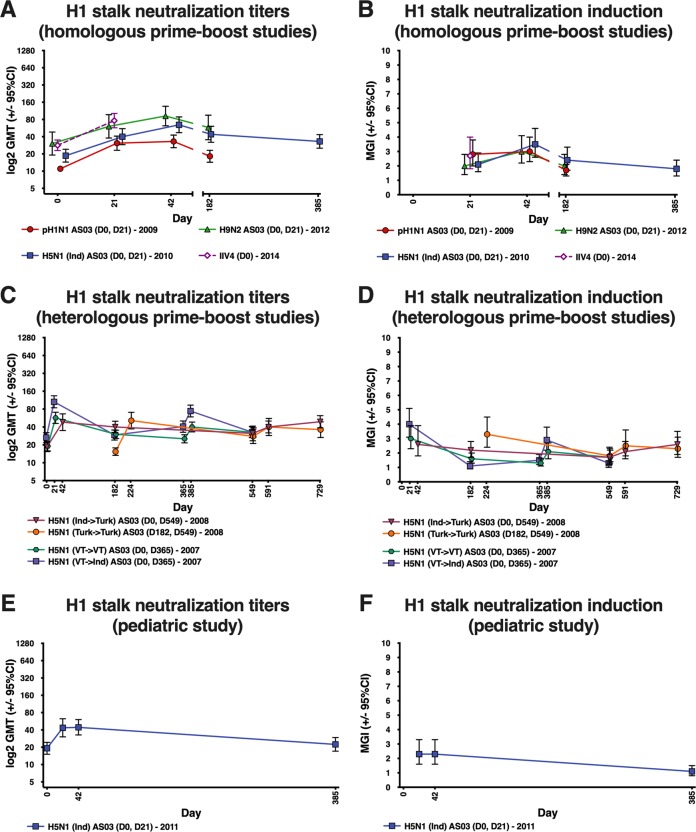
GMTs and MGIs for anti-H1 stalk antibodies measured by microneutralization assay following vaccination with adjuvanted pandemic vaccines and seasonal vaccine. Ind: A/Indonesia/5/05; Turk: A/turkey/Turkey/1/2005; VT: A/Vietnam/1194/2004. Error bars indicate 95% confidence intervals. GMT: geometric mean titer; IIV4: inactivated quadrivalent influenza vaccine; MGI: mean geometric increase.

For all vaccines, some level of neutralizing anti-H1 stalk antibodies was observed (Fig. 3a–f). For vaccine heterosubtypic antibodies, post-vaccination GMTs and MGIs were generally low against A/H5N8, A/H1N1 avian-like swine influenza virus and A/H1N1pdm09 virus in the A/H5N1 and A/H9N2 groups, and against A/H5N8 in the A/H1N1 and IIV4 groups (Supplementary Figs. 2, 3 and 4). However, higher neutralizing titers were induced against A/H1N1 avian-like swine influenza virus and A/H1N1pdm09 virus in the A/H1N1 and IIV4 groups.

### Plasmablast and memory B-cell response

**Fig. 4:**
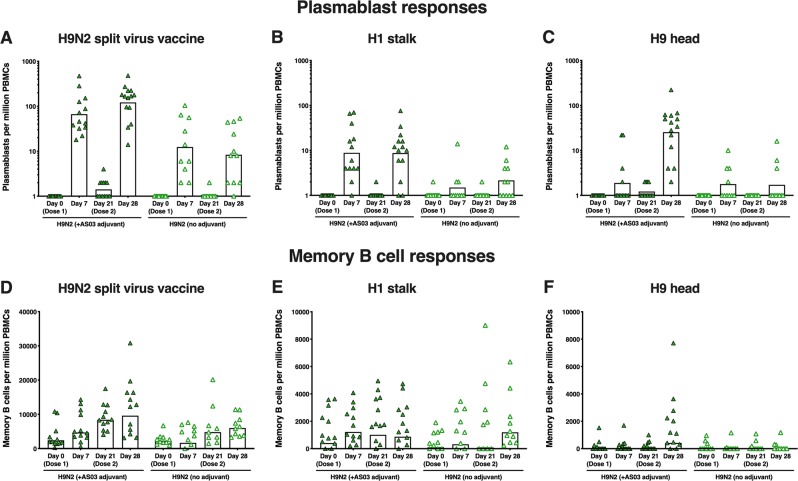
Plasmablast and memory B-cell responses against H9N2 split virus, H1 stalk domain and H9 head domain following vaccination with H9N2 vaccine.

The kinetics of plasmablast detection in peripheral blood cells were as expected,^33,34^ peaking 1 week post-vaccination. Plasmablast responses against the A/H9N2 split virus and the H1 stalk domain (cH6/1) were detected 7 days after both the first and second vaccine dose, with a trend for a higher response after the second dose (Fig. 4a–c). By contrast, plasmablast responses to the H9 head domain were scarce after the first dose, but were induced in most participants after the second adjuvanted dose, indicating that the homologous booster re-established the immunodominance of the HA head domain (Fig. 4c). The adjuvanted vaccine elicited stronger plasmablast responses than the non-adjuvanted vaccine. The memory B-cell response against the A/H9N2 split virus and the H1 stalk domain followed the same kinetic as the cognate plasmablast response (Fig. 4d–f).

### Passive transfer/viral challenge

The degree of protection in mice offered by antibodies in the serum of vaccinees who received AS03-adjuvanted A/H5N1 vaccine (trial 2) against challenge with a lethal dose of cH6/1N5 or cH5/3N4 virus was evaluated. Since humans are naive for the H6 head and N5, the only antigen in the cH6/1N5 virus against which humans would be primed is the H1 stalk; thus, challenge with the cH6/1N5 virus predominantly measured H1 HA stalk antibodies. On the same principle, challenge with the cH5/3N4 virus predominantly measured HA head antibodies.

**Fig. 5:**
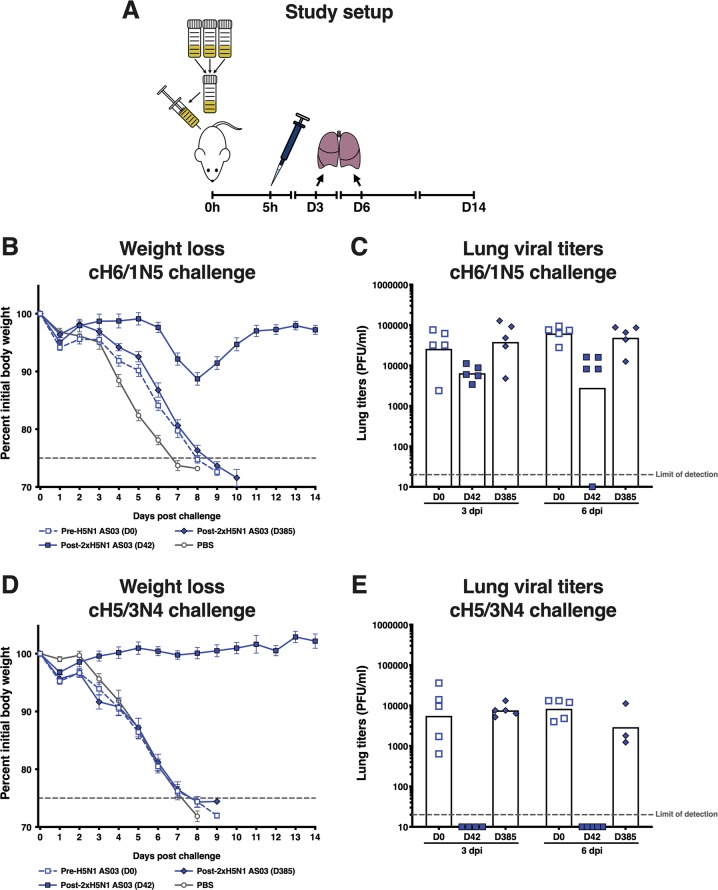
Protection offered by serum from AS03-adjuvanted H5N1 vaccinees in mice challenged with lethal doses of cH6/1N5 viruses (to assess stalk domain protection) or cH5/3N4 viruses (to assess head domain protection). Error bars indicate standard error of the mean (SEM). D: Day; dpi: days post injection; PBS: phosphate-buffered saine; PFU: plaque-forming units.

Serum collected at 42 days post-vaccination was protective against weight loss induced by challenge with cH6/1N5 virus and reduced viral lung titers (Fig. 5a, b), while serum collected at baseline and 1 year after vaccination did not. Even greater protection against weight loss and very low viral lung titers were observed following challenge with the cH5/3N4 head domain virus in mice that received serum collected 42 days post-vaccination (Fig. 5d, e). Mice that received human serum collected at baseline and 1 year after vaccination suffered substantial weight loss and had high viral lung titers following challenge with cH5/3N4 virus.

## Discussion

Targeting the highly conserved HA stalk domain via sequential immunization with vaccines containing a chimeric HA composed of an exotic head and conserved stalk domain is one approach towards development of a universal influenza vaccine. Although the HA head domain is immunodominant, this strategy may redirect the immune system towards the stalk domain.^16,17^ Adult humans have a highly complex and variable exposure history to influenza virus antigens either through natural exposure to circulating stains and/or seasonal vaccination. In theory, pre-existing anti-stalk antibodies induced by these previous experiences should be boosted by immunization with a pandemic influenza vaccine that contains HA from an influenza virus strain not circulating in humans because the head domain will not be recognized and memory B-cells that target conserved epitopes in the stalk domain will be activated instead. In the present study, we tested the hypothesis that pandemic influenza vaccination could act as a surrogate for one dose of a chimeric HA vaccine and boost an immune response against the stalk domain in a primed population.

**Fig. 6:**
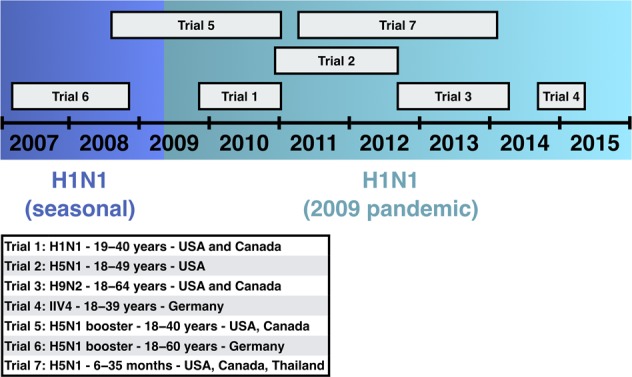
Timelines of the clinical trials.

All adults in our study were ELISA-positive for anti-H1 stalk antibodies pre-vaccination, consistent with previous studies showing that adults have pre-existing HA stalk antibodies.^21,35^ Pre-vaccination GMTs in adults were generally consistent across trials and ranged between 7000−14,000 EU/mL. Minor differences in pre-vaccination titers between trials might be partially explained by different trial inclusion/exclusion criteria, including participant age, or by the difference in prior virus exposure between trials. Some of the trials were performed before and others after the 2009 A/H1N1 pandemic (Fig. 6), and it has been previously shown that titers of HA stalk antibodies were boosted following the pandemic.^36,37^

We observed that titers of anti-H1 stalk antibodies rose substantially following the first dose of A/H1N1, A/H5N1 and A/H9N2 vaccines, in line with similar studies in which induction of anti-H1 stalk serum antibodies was evaluated in people receiving an A/H5N1 or A/H7N9 vaccine.^22–24^ Induction, as measured by MGI, was higher with A/H1N1 vaccination than with A/H5N1 or A/H9N2 vaccination, possibly resulting from lower pre-vaccination titers in the A/H1N1 group. The adjuvant effect was variable between the vaccines; the A/H1N1 vaccine elicited a strong immune response with or without adjuvant, whilst a strong adjuvant effect was observed for the A/H5N1 and A/H9N2 vaccines. This may be explained by differences in the immunogenicity of the vaccine virus strain or by differences between study groups in the level of pre-existing antibody titers. Anti-H1 stalk antibody titers remained higher than pre-vaccination levels 6 months after A/H1N1, A/H5N1 and A/H9N2 vaccination (and 1 year after A/H5N1 vaccination), particularly in the adjuvanted groups. Vaccination with seasonal IIV4 (non-adjuvanted) also induced anti-H1 stalk antibodies. In a separate study, we observed that mice serially vaccinated with monovalent A/H1N1, A/H5N1 and A/H9N2 influenza virus vaccines also showed induction of HA stalk antibodies, with higher antibody levels seen in groups that received adjuvanted vaccines. Data and brief methodology for this study are described in Supplementary Fig. 5.

All children were ELISA-positive for anti-H1 stalk antibodies pre-vaccination, albeit at much lower levels than adults. Following vaccination with A/H1N1, A/H5N1 and A/H9N2 vaccines, titers of anti-H1 stalk antibodies were lower in children than in adults but the pattern of the response was similar. Vaccination of young children with no prior exposure to influenza virus by infection or vaccination might require specific vaccination strategies to initially prime for HA stalk antibodies, before aiming to elicit high antibody titers using universal influenza virus vaccine antigens. In preclinical studies, priming with inactivated seasonal influenza virus vaccines has shown some promise.^19,38^ It might be possible to similarly prime children with a seasonal influenza virus vaccine, before using universal vaccine antigens.

In the homologous prime-boost studies, with a 21-day prime-boost schedule (trials 1−3 and 7), the second vaccine dose induced limited anti-stalk antibodies. This was expected, as the response is likely to be directed towards the HA head domain rather than to the HA stalk. Indeed, this was demonstrated in the present study with the induction of an H9 head-specific plasmablast response after the second dose of the A/H9N2 vaccine. Trials 5 and 6 compared heterologous booster regimens (A/H5N1 Indonesia prime followed by A/H5N1Turkey boost and A/H5N1 Vietnam prime followed by A/H5N1 Indonesia boost) versus homologous booster regimens (two doses of A/H5N1 Turkey or two doses of A/H5N1 Vietnam). The objective of the comparison was to evaluate whether heterologous, but homosubtypic, strains of A/H5N1 would be sufficiently distinct to predominantly boost HA stalk antibodies instead of HA head-specific responses. Anti-stalk antibody titers rose after the priming dose, fell to just above baseline levels in the 12−18 months before administration of the booster dose, then rose following the booster dose to levels similar to those induced by the priming dose. The titers induced by the booster dose were similar regardless of whether a heterologous or homologous booster dose was administered. In the heterologous prime-boost regimens, the head domain in both the priming and booster vaccines was similar. The booster dose may therefore have boosted head responses, rather than eliciting further rises in anti-stalk titers. This underlines the requirement for sequential vaccination with very different head domains or a headless HA stalk as part of the universal vaccine strategy.

We evaluated the breadth of the immune response to adjuvanted pandemic vaccines and the IIV4 using full length recombinant H2 and H18 HA antigens. We chose H2 because it is from the same clade as the H1 virus and has previously caused a pandemic in humans.^39^ H18 was selected because it is from the most divergent clade to H1 within the influenza A virus group 1 HAs. A post-vaccination increase in anti-H2 and anti-H18 GMTs was observed in all groups, indicating that some antibodies induced by pandemic influenza virus vaccines can be cross-reactive to viruses within the group 1 HAs. In our previous study, we found cross-reactivity to both H2 and H18 viruses, but not to the group 2 H3 virus.^22^ Therefore, a stalk-based universal influenza virus vaccine is likely to need either HA antigens from both influenza A virus group 1 and group 2, or a stalk domain that induces antibodies against both groups, as well as influenza B virus stalk antigens.

Some induction of neutralizing anti-stalk antibodies was observed in all studies, although at a lower level than the neutralizing antibodies against the vaccine-homologous strain induced in the primary A/H5N1 and A/H9N1 studies (trials 2 and 3).^26,27^ Weaker induction of neutralizing anti-stalk antibodies is consistent with the low neutralizing activity described for anti-HA stalk antibodies, which have been previously shown to mediate protection mainly via Fc-mediated functions.^39–43^ Vaccine heterosubtypic neutralization was low in the A/H5N1 and A/H9N2 vaccine groups. However, vaccination with A/H1N1 and IIV4 vaccines induced high neutralizing titers against A/H1N1 avian-like swine influenza virus and A/H1N1pdm09 influenza virus. This effect is likely to be driven by conservation of an antigenic site in the head domain.

After the first vaccine dose in the A/H9N2 vaccine group, a strong plasmablast response was seen against the A/H9N2 split virus and the H1 stalk domain (albeit at lower frequencies), but not against the trimeric H9 head domain. Similar frequencies of plasmablast response against the split virus and the stalk domain were recalled following the second vaccine dose (following a similar pattern to stalk-specific antibody response), and a response against the H9 head domain was also observed (in contrast to after the first dose). This is consistent with previous data generated with A/H5N1 pandemic vaccine showing that the second vaccine dose (re)directs the immune response against the head domain.^23^ Plasmablast responses were higher in the AS03-adjuvanted group than in the non-adjuvanted group. We observed an increase in memory B-cell response to A/H9N2 split virus and the H1 stalk domain after the first and second vaccine doses, but against the H9 head only after the second dose of AS03-adjuvanted vaccine.

We assessed whether antibodies in the serum of individuals who received A/H5N1 vaccine (trial 2) would offer protection in vivo to mice against challenge with a lethal dose of cH6/1N5 or cH5/3N4 virus. The cH6/1N5 virus challenge predominantly measures anti-H1 stalk antibodies; because humans are naive for the H6 head and for N5, the H1 stalk is the only HA antigen in this virus against which humans would be primed. Likewise, the cH5/3N4 virus challenge predominantly measures anti-H5 head antibodies. We found that serum collected pre-vaccination had no protective effect in the chosen set-up, but serum collected 42 days after vaccination with AS03-adjuvanted A/H5N1 prevented weight loss and reduced virus lung titers. The serum appeared to offer complete protection against challenge with cH5/3N4 at five times the LD_50_ dose, in that mice exhibited no morbidity and there was a near absence of viral replication, suggesting that H5 head responses completely neutralized the virus. Protection against cH6/1N5 challenge (also at five times the LD_50_) was less complete (some weight loss was observed before recovery), but was still very substantial compared with pre-vaccination serum. Viral lung titers were reduced, indicating some infection-modifying activity of the H1 stalk responses.

A protective effect of anti-stalk antibodies induced by an A/H5N1 vaccine against challenge with a viral strain expressing H1 stalk has been previously described.^40^ The differences between the protective effect against the two challenge strains likely highlight different mechanisms of action for the two kinds of antibodies (HA head-specific versus HA stalk-specific). However, it is not possible to directly compare the level of protection provided by the HA head and HA stalk antibodies in this experiment because the viruses display different phenotypes in mice. Overall, the cH6/1N5 virus was more lethal at low doses than the cH5/3N4 virus, indicating a fitter virus, as reflected in the difference between lung viral titers in mice that received serum collected pre-vaccination. We therefore cannot conclude that anti-HA head antibodies provide superior protection compared with anti-HA stalk antibodies. Serum collected 1 year after vaccination failed to provide protection against either challenge strain (cH6/1N5 and cH5/3N4 viruses). This suggests that in this preclinical model, the anti-HA head response induced by the adjuvanted A/H5N1 vaccine did not outperform the anti-HA stalk response with regard to long-term protection against a lethal challenge dose. It should be noted that, if humans with pre-existing antibodies were exposed to a similar challenge, the antibody-producing cells corresponding to these antibodies would respond by producing more antibody to combat the infection. This could result in faster clearance of virus in humans than was observed in these otherwise naive mice.

Chimeric HA-based influenza virus vaccine candidates are currently being tested in clinical trials. A randomized, Phase I trial is evaluating the immunogenicity of regimens comprising initial vaccination with a cH8/1N1 live attenuated vaccine followed by adjuvanted and non-adjuvanted cH5/1N1 inactivated vaccine, or initial vaccination with a cH8/1N1 adjuvanted inactivated vaccine followed by adjuvanted cH5/1N1 inactivated vaccine (NCT03300050), and a Phase I/II trial is evaluating the immunogenicity of nine formulations of adjuvanted and non-adjuvanted chimeric HA inactivated vaccines (NCT03275389). An interim analysis of the trial using the cH8/1N1 construct demonstrated that prime vaccination with the inactivated vaccine, but not the live attenuated vaccine, induced an immune response, whilst all regimens induced an immune response after boost vaccination.^44^ While we have previously shown in a preclinical mouse model that HA stalk antibody responses can be maintained at high levels for long durations,^19^ the antibody titers elicited in the Phase I trial started to wane after initial strong increases.^44^ This could be due to pre-existing B cells that differentiated into short-lived plasmablasts, but not long-lived plasma cells. However, HA stalk antibody titers were maintained at levels above baseline in individuals who received adjuvanted vaccines.^44^ To further improve a sustained high antibody response, it will be important to identify approaches that can preferentially induce long-lived plasma cells that would continuously produce HA stalk antibodies.

In conclusion, our study showed that immunization with A/H1N1, A/H5N1 and A/H9N2 pandemic vaccines expressing HA head domains, for which vaccinees lack immunity, induced anti-H1 stalk immune responses in adults and children. Anti-H1 stalk antibodies were present before vaccination in adults at higher levels than in children. The effect of the AS03 adjuvant was variable. The antibodies induced after vaccination were cross-reactive to H2 and H18 viruses, indicating heterosubtypic immunity. Furthermore, sera from vaccinees provided an in vivo protective effect in mice, illustrating antibody functionality most likely through the Fc region despite low titers of neutralizing antibodies. These findings support the concept of a universal vaccine strategy based on induction of anti-stalk antibodies via sequential vaccination with different HA head domains.

## Methods

### Ethics

This was a retrospective study that used archived serum samples from seven completed clinical trials. The study protocol was approved by an Independent Ethics Committee at Hôpital Erasme (Université libre de Bruxelles; Protocol # P2015/173) and the study was conducted in accordance with the Declaration of Helsinki and Good Clinical Practice. Participants or legally acceptable representatives provided written informed consent to participate in the original clinical trials and consent for their samples to be used in further research. This study was registered with clinicaltrials.gov (NCT02415842) on April 14, 2015.

### Clinical trials, participants, and vaccines

We included data from the following trials of pandemic or pre-pandemic influenza virus vaccines (hereafter referred to as pandemic influenza vaccines for convenience) or seasonal influenza virus vaccine: three homologous prime-boost trials in adults with inactivated, split-virion A/H1N1, A/H5N1 and A/H9N2 pandemic influenza vaccines (trials 1, 2 and 3), one trial with a single dose of seasonal inactivated quadrivalent influenza virus vaccine (IIV4) in adults (trial 4), two heterologous prime-boost trials with A/H5N1 pandemic influenza vaccines in adults (trials 5 and 6), and one homologous prime-boost trial with A/H5N1 pandemic influenza vaccine in children (trial 7) (Fig. 6; Table 1; Supplementary Table 6).^25–32^ We aimed to select approximately 30 participants from the relevant treatment groups of each trial. Participants were eligible for the present study if they had completed the clinical trial according to the protocol and had sufficient residual sample volume at all protocol-specified sample timepoints.

All vaccines were manufactured in an egg-based system with the exception of the A/H5N1 vaccine used in trial 2, which was cell culture-derived. The vaccines (0.5 mL volume) were administered in the deltoid muscle or the thigh for children <12 months of age. Vaccine strains, vaccination schedule and blood sampling schedule for immune response measurements are described in Table 1. AS03 is an oil-in-water emulsion-based Adjuvant System containing ɑ-tocopherol (AS03_A_: 11.86 mg; AS03_B_: 5.93 mg).^45^

### Study objectives

We included trials 1−4 to test the potential of pandemic influenza virus vaccines with exotic HA head domains to elicit anti-HA stalk antibodies in individuals naïve to these strains compared with a seasonal influenza virus vaccine, which is thought to predominantly elicit anti-HA head-specific immune responses due to prior exposure to homosubtypic seasonal strains. Trials 5 and 6 tested whether heterologous strains of the same virus subtype (A/H5N1) would be sufficiently distinct to predominantly boost HA stalk antibodies instead of HA head-specific responses. We included the pediatric cohort (trial 7) to test whether HA stalk antibodies can be elicited in individuals with very low pre-existing HA stalk antibody levels.

We selected assays and antigens to evaluate the level and functionality of anti-stalk antibodies elicited by the pandemic vaccines, as well as the breath of the immune response. In addition, we evaluated cell-mediated immunity in terms of the B-memory cell and plasmablast response, as well as the in vivo protective effect in mice of serum from trial participants.

The study had four co-primary objectives, to measure: (1) anti-H1 stalk antibody response by enzyme-linked immunosorbent assay (ELISA); (2) anti-H1 stalk antibody response by microneutralization (MN) assay in participants who received an AS03-adjuvanted vaccine or the IIV4; (3) anti-H2 full length HA and anti-H18 full length HA antibody response by ELISA in participants who received an AS03-adjuvanted vaccine or the IIV4; and (4) vaccine-heterosubtypic antibody response by MN assay in participants who received an AS03-adjuvanted vaccine. The antibody response was summarized in terms of geometric mean titers (GMT) and the mean geometric increase (MGI).

We evaluated the effect of the AS03 adjuvant on levels of anti-stalk antibody by ELISA as a secondary objective in terms of the GMT ratio (AS03-adjuvanted/non-adjuvanted) and the difference in the percentage of participants with ≥4-fold rise in titer (AS03-adjuvanted minus non-adjuvanted). We also measured pre-vaccination seropositivity rate by HI assay to pandemic vaccine homologous virus as a secondary objective.

We evaluated cell-mediated immunity as a tertiary endpoint in terms of the B-memory cell and plasmablast response among participants in trial 3 (the only trial in which these cells were collected). In addition, a serum passive transfer/viral challenge experiment evaluated the in vivo protective effect in mice of samples from participants in trial 2 who received AS03-adjuvanted vaccine.

### Immunogenicity assays

#### ELISA

We measured anti-H1 stalk antibodies using a recombinant chimeric cH6/1 HA antigen with the H6 head domain from A/mallard/Sweden/81/2002 (A/H6N1) and H1 stalk domain from A/California/04/2009 (A/H1N1 pandemic strain) (Table 2). In addition, we evaluated the breadth of the immune response using full length recombinant H2 and H18 HA antigens, based on A/Japan/305/1957 and A/flat-faced bat/Peru/033/2010 viruses, respectively^46,47^ (Table 2). The recombinant proteins were expressed in *Trichoplusia ni* derived BTI-TN-5B1-4 cells (High Five), using a baculovirus expression system. All proteins contained a C-terminal trimerization domain and a hexahistidine tag used for purification. We used a classical ELISA in which the antigen was coated on 96-well plates, and, after blocking, the serum was added and sequentially diluted. After incubation and washing steps, a detection antibody (Mouse anti-Human IgG HRP clone JDC-10 [Southern Biotech, cat. no. 9040-05]; 1:2000) was used to distinguish serum antibodies attached to the antigen. Serum antibodies were quantified by optical density measurements. Positive and negative controls were developed in addition to an antigen-specific standard. The assay cut-off was 66 EU/mL (ELISA Units/mL).

**Table 2:** Antigens used in immunogenicity assays.

Endpoint/objective	Antigen	Assay/test	Trial and vaccine
Anti-H1 stalk antibody titers	Chimeric cH6/1HA antigen: A/mallard/Sweden/81/2002 (A/H6N1) A/California/04/2009 (A/H1N1pdm09)	ELISA	All trials and vaccines
Anti-H1 stalk antibody neutralization	Reverse genetics reassortant virus with H6 head, H1 stalk and N5: A/mallard/Sweden/81/2002 (A/H6N1) A/California/04/2009 (A/H1N1pdm09) A/mallard/Sweden/86/2003 (A/H12N5)	MN	Adjuvanted pandemic vaccines and IIV4
Breadth of immune response	Full length H2 antigen: A/Japan/305/1957	ELISA	Adjuvanted pandemic vaccines and IIV4
	Full length H18 antigen: A/flat-faced bat/Peru/033/2010		
Vaccine-heterosubtypic neutralization	Reverse genetics reassortant virus: A/gyrfalcon/Washington/41088-6/2014 (A/H5N8)	MN	Adjuvanted pandemic vaccines and IIV4
	A/H1N1 avian-like swine influenza virus: A/swine/Jiangsu/40/2011		
	A/H1N1pdm09 influenza virus: A/Singapore/GP1908/2015		
Memory B-cell response Plasmablast response	A/H9N2 split virus: A/chicken/Hong Kong/G9/1997	ELISpot	Trial 3 (adjuvanted and non-adjuvanted A/H9N2 vaccine)
	Chimeric cH6/1HA antigen: A/mallard/Sweden/81/2002 (A/H6N1) A/California/04/2009 (A/H1N1pdm09)		
	Trimeric H9 head domain based on: A/chicken/Hong Kong/G9/1997		
In vivo protection in mice	Chimeric cH6/1HA antigen: A/mallard/Sweden/81/2002 (A/H6N1) A/California/04/2009 (A/H1N1pdm09)	Weight loss Lung virus titer	Trial 2 (adjuvanted A/H5N1 vaccine)
	Reverse genetics reassortant virus with H6 head, H1 stalk and N5: A/mallard/Sweden/81/2002 (A/H6N1) A/California/04/2009 (A/H1N1pdm09) A/mallard/Sweden/86/2003 (A/H12N5)		

*ELISA* enzyme linked immunosorbent assay; *MN* microneutralizing

#### MN assay

We evaluated the functionality of the anti-H1 stalk antibodies by MN assay using a reverse genetics reassortant virus with the H6 head domain from A/mallard/Sweden/81/2002 (A/H6N1), H1 stalk domain from A/California/04/2009 (A/H1N1 pandemic strain) and N5 from A/mallard/Sweden/86/2003 (A/H12N5) (Table 2). Since humans are generally naïve to the H6 head domain and the N5 neuraminidase, this virus should primarily measure HA stalk antibody mediated neutralization. Vaccine-heterosubtypic neutralization was evaluated using the same method for A/H5N8 (reverse genetics reassortant virus with HA and NA from A/gyrfalcon/Washington/41088-6/2014), A/H1N1 avian-like swine influenza virus (A/swine/Jiangsu/40/2011) and A/H1N1pdm09 virus (A/Singapore/GP1908/2015) (Table 2).

Samples were treated with receptor-destroying enzyme (RDE) (Denka Seiken) and heat inactivated for 30 min at 56 °C. A standardized amount of virus (200 plaque-forming units [PFU] or 100 times the 50% tissue culture infective dose, depending on the virus strain) was mixed with serial dilutions of serum in N-tosyl-L-phenylalanine chloromethyl ketone-treated trypsin-containing UltraMDCK media (Lonza Bioscience) (1:1000 dilution) and incubated to allow binding of the antibodies to the virus for 1 h at room temperature. The virus-serum mixture was added onto Madin-Darby canine kidney cells and incubated at 33 °C or 37 °C (depending on the virus strain) for 1 h. After the incubation period, the virus-serum mixture was removed and replaced with diluted serum at the previous concentration. After an incubation period of 48−72 h (depending on the virus strain), virus replication was visualized by measuring the hemagglutination of chicken red blood cells (concentration: 0.5%) by the cell supernatant and a neutralization titer was calculated at the highest serum dilution able to totally neutralize the virus. Each serum sample was tested once. The assay cut-off was 1:10 DIL.

#### Hemagglutination inhibition assay

HI assays were performed against the matched vaccine strains. Measurements were conducted on thawed serum samples with a standardized and comprehensively validated micro-method using two hemagglutinating units of the appropriate antigens per 25 µL and a 0.45% fowl erythrocyte suspension.^48^ Non-specific serum inhibitors were removed by treatment with RDE and heat inactivation. Starting with an initial dilution of 1:10, a dilution series (by a factor of 2) was prepared up to an end dilution of 1:10,240. The titration endpoint was taken as the highest dilution step that showed complete inhibition of hemagglutination. All assays were performed in duplicate. The cut-off value was 1:10 DIL.

#### Memory B-cell and plasmablast detection assays

We used the enzyme-linked immunospot (ELISpot) assay to evaluate the frequency (per million memory B-cells) of HA stalk-specific memory B-cells from peripheral blood samples. The assay was based on the method of Crotty (2004)^49^ in which peripheral blood mononuclear cells (PBMCs) were stimulated for 5 days in vitro to allow differentiation of memory B-cells into antibody secreting cells, followed by incubation in nitro-plates coated with either the antigen of interest (for the detection of antigen-specific memory B-cells) or anti-human Ig (for the detection of total memory B-cells). A conventional immuno-enzymatic procedure was applied to detect antibody/antigen spots enumerating memory B-cells and the results were expressed as the frequencies of antigen-specific memory B-cells within the total memory B-cell population. The same method without the in vitro stimulation step was applied to measure the plasmablast response. The response to the following antigens was evaluated: A/chicken/Hong Kong/G9/1997 (A/H9N2) split virus, H1 stalk domain (chimeric cH6/1 antigen as described above) and trimeric H9 globular head domain (recombinant protein based on A/chicken/Hong Kong/G9/1997) (Table 2).

#### Passive transfer/viral challenge

Serum samples collected from participants in trial 2 who received AS03-adjuvanted vaccine were pooled by timepoint (baseline, 42 days after first vaccination [Day 42] and 1 year after vaccination [Day 385]). We included baseline data to allow comparison of the protective effect achieved with pre-vaccination versus post-vaccination serum. Recipient BALB/c mice were injected intraperitoneally with 150 µL of pooled serum and subsequently challenged with cH5/3N4 or cH6/1N5 virus (measuring head and stalk responses, respectively) (Table 2). The level of protection offered by the serum was assessed in terms of mean weight loss (change from baseline over 14 days post challenge) and change in lung virus titer (log_10_ fold difference versus baseline [PFU/mL]). Fifty microliters of virus preparation diluted in phosphate buffer saline was administered intranasally. The cH5/3N4 virus was given at a dose of 16,000 PFU for analysis of weight loss (equivalent to 5xLD_50_ in the presence of normal human serum) and 280 PFU for analysis of lung virus titer. Corresponding doses for cH6/1N5 virus were 200 PFU (equivalent to 5xLD_50_ in the presence of normal human serum) and 18 PFU. Ten mice were included in each group. Mice that lost >25% of their initial body weight following viral challenge were euthanized for ethical reasons and considered as mortalities.

### Statistical methods

The GMT calculations were performed by taking the anti-log of the mean of the log10 titer transformations. Antibody titers below the assay cut-off were given an arbitrary value of half the cut-off for the GMT calculation. The 95% confidence intervals (CI) for GMTs were obtained within each group separately. The 95% CI for the mean of log-transformed titer was first obtained assuming that log-transformed values were normally distributed with unknown variance. The 95% CI for the GMTs was then obtained by exponential-transformation of the 95% CI for the mean of log-transformed titer. All computed CIs were two-sided. The exact 95% CIs for a proportion within a group were based on the method by Clopper and Pearson.^50^ The adjusted GMT ratios and 95% CIs for the comparison of adjuvanted with non-adjuvanted groups were computed using ANCOVA models on the log10 transformation of the titers, including the vaccine group as fixed effect and the baseline titer as covariate.

The study was descriptive. Each group and trial was reported separately.

## Supplementary information

Supplemental Material

## Data Availability

GSK makes available anonymized individual participant data and associated documents from interventional clinical studies which evaluate medicines, upon approval of proposals submitted to www.clinicalstudydatarequest.com. To access data for other types of GSK sponsored research, for study documents without patient-level data and for clinical studies not listed, please submit an enquiry via the website.
