# Supplementary material for: Pandemic influenza virus vaccines boost hemagglutinin stalk-specific antibody responses in primed adult and pediatric cohorts
Source: NPJ Vaccines. 2019 Dec 6;4:51. doi: 10.1038/s41541-019-0147-z (PMC6898674; doi:10.1038/s41541-019-0147-z)
Supplement: Supplementary file 1 — Supplemental Material [file 41541_2019_147_MOESM1_ESM.pdf]

# Supplementary information

R Nachbagauer et al: Pandemic influenza virus vaccines boost hemagglutinin stalk-specific antibody responses in primed adult and pediatric cohorts

**Supplementary Table 1. Baseline demographics**

| Trial | Study group | N | Age, y Mean (SD) | Female n (%) | Geographic ancestry n (%) | | | |
|---|---|---|---|---|---|---|---|---|
| | | | | | African or African American | White | Asian | Other |
| 1 Adults | H1N1 AS03 | 29 | 28.5 (6.5) | 19 (65.5) | 3 (10.3) | 25 (86.2) | 1 (3.4) | 0 |
| | H1N1 non-adjuvanted | 29 | 29.3 (6.8) | 18 (62.1) | 2 (6.9) | 27 (93.1) | 0 | 0 |
| 2 Adults | H5N1 AS03 | 29 | 30.4 (8.3) | 17 (58.6) | 7 (24.1) | 19 (65.5) | 2 (6.8) | 1 (3.4) |
| | H5N1 non-adjuvanted | 27 | 31.4 (9.1) | 18 (66.7) | 7 (25.9) | 19 (70.4) | 1 (3.7) | 0 |
| 3 Adults | H9N2 AS03 | 30 | 43.3 (11.9) | 14 (46.7) | 3 (10.0) | 27 (90.0) | 0 | 0 |
| | H9N2 non-adjuvanted | 30 | 42.6 (13.6) | 16 (53.3) | 4 (13.3) | 23 (76.7) | 1 (3.3) | 2 (6.7) |
| 4 Adults | IIV4 non-adjuvanted | 30 | 26.7 (5.7) | 22 (73.3) | 0 | 30 (100) | 0 | 0 |
| 5 Adults | H5N1 AS03 Indonesia > Turkey | 26 | 29.4 (6.5) | 12 (46.2) | 3 (11.5) | 22 (84/6) | 0 | 1 (3.8) |
| | H5N1 AS03 Turkey > Turkey | 29 | 29.6 (6.8) | 16 (55.2) | 6 (20.7) | 22 (75.9) | 1 (3.4) | 0 |
| 6 Adults | H5N1 AS03 Vietnam > Vietnam | 53 | 34.8 (12.1) | 21 (39.6) | 0 | 53 (100) | 0 | 0 |
| | H5N1 AS03 Vietnam > Indonesia | 49 | 35.4 (12.5) | 28 (57.1) | 0 | 49 (100) | 0 | 0 |
| 7 Children | H5N1 AS03 | 33 | 22.6 (7.6)* | 16 (48.5) | 4 (12.1) | 10 (30.3) | 19 (57.6) | 0 |
| | Placebo | 20 | 23.5 (6.9)* | 10 (50.0) | 0 | 7 (35.0) | 12 (60.0) | 1 (5.0) |

SD: standard deviation

*Age in months

**Supplementary Table 2. Pre-vaccination seropositivity for HI against the vaccine-homologous virus and the A/H1N1pdm09 virus**

| Trial | Study group | N | n (%) | | | | | |
|---|---|---|---|---|---|---|---|---|
| | | | **A/California/7/ 2009*** | **A/Indonesia/5/ 2005** | **A/chicken/Hong Kong/G9/1997** | **A/Christchurch/16/ 2010** | **A/Turkey/01/ 2005** | **A/Vietnam/1194/ 2004-like** |
| 1 Adults | A/H1N1 AS03 | 29 | 12 (41.4) | - | - | - | - | - |
| | A/H1N1 non-adjuvanted | 29 | 10 (34.5) | - | - | - | - | - |
| 2 Adults | A/H5N1 AS03 | 29 | 21 (75.0) | 9 (31.0) | - | - | - | - |
| | A/H5N1 non-adjuvanted | 27 | 18 (66.7) | 1 (3.7) | - | - | - | - |
| 3 Adults | A/H9N2 AS03 | 30 | 26 (86.7) | - | 11 (36.7) | - | - | - |
| | A/H9N2 non-adjuvanted | 30 | 23 (76.7) | - | 12 (40.0) | - | - | - |
| 4 Adults | IIV4 non-adjuvanted | 30 | - | - | - | 27 (90.0) | - | - |
| 5 Adults | A/H5N1 AS03 Indonesia > Turkey | 26 | 10 (38.5) | 0 | - | - | 1 (3.8) | - |
| | A/H5N1 AS03 Turkey > Turkey | 29 | 14 (48.3) | - | - | - | 5 (17.2) | - |
| 6 Adults | A/H5N1 AS03 Vietnam > Vietnam | 53 | 19 (38.0) | - | - | - | - | 1 (1.9) |
| | A/H5N1 AS03 Vietnam > Indonesia | 49 | 15 (31.9) | 0 | - | - | - | 0 |
| 7 Children | A/H5N1 AS03 | 33 | - | 0 | - | - | - | - |
| | Placebo | 20 | - | - | - | - | - | - |

*A/California/7/2009 was the A/H1N1pdm09 virus and the vaccine-homologous virus in Trial 1

N: number of evaluable participants; n: number of seropositive participants (antibody titer ≥10 1/DIL)

**Supplementary Table 3. GMTs and MGIs for anti-H1 stalk antibodies measured by ELISA following vaccination with adjuvanted and non-adjuvanted pandemic vaccines and seasonal vaccine**

| Trial | Study group | Time point | GMT (95% CI) | | | | MGI [post-vaccination/pre-vaccination] (95% CI) | | | |
|---|---|---|---|---|---|---|---|---|---|---|
| | | | N | Point estimate | LL | UL | N | Point estimate | LL | UL |
| 1 | A/H1N1 AS03 | Pre | 27 | 6935.6 | 4974.4 | 9670.0 | - | - | - | - |
| | | D21 | 28 | 45098.3 | 34775.1 | 58486.2 | 27 | 6.5 | 4.7 | 9.2 |
| | | D42 | 27 | 46177.1 | 37510.4 | 56846.2 | 25 | 7.0 | 5.0 | 9.7 |
| | | D182 | 29 | 21782.4 | 17803.2 | 26651.0 | 27 | 3.2 | 2.4 | 4.1 |
| | A/H1N1 non-adjuvanted | Pre | 25 | 6841.5 | 4954.4 | 9447.5 | - | - | - | - |
| | | D21 | 25 | 37969.9 | 26930.8 | 53534.1 | 23 | 5.9 | 3.6 | 9.6 |
| | | D42 | 27 | 38598.2 | 27818.0 | 53555.9 | 25 | 5.6 | 3.6 | 8.7 |
| | | D182 | 29 | 24705.6 | 18369.2 | 33227.7 | 25 | 3.6 | 2.4 | 5.3 |
| 2 | A/H5N1 AS03 | Pre | 29 | 14381.9 | 10313.7 | 20054.7 | - | - | - | - |
| | | D21 | 29 | 46013.8 | 33592.9 | 63027.5 | 29 | 3.2 | 2.3 | 4.5 |
| | | D42 | 29 | 68348.8 | 53244.4 | 87738.1 | 29 | 4.8 | 3.5 | 6.4 |
| | | D182 | 29 | 33610.2 | 26464.5 | 42685.3 | 29 | 2.3 | 1.8 | 3.1 |
| | | D385 | 29 | 25346.9 | 20205.0 | 31797.3 | 29 | 1.8 | 1.4 | 2.2 |
| | A/H5N1 non-adjuvanted | Pre | 27 | 14552.3 | 10670.0 | 19847.2 | - | - | - | - |
| | | D21 | 27 | 27845.9 | 20269.3 | 38254.5 | 27 | 1.9 | 1.4 | 2.7 |
| | | D42 | 27 | 31718.0 | 23755.4 | 42349.5 | 27 | 2.2 | 1.6 | 3.0 |
| | | D182 | 27 | 17401.9 | 13330.1 | 22717.5 | 27 | 1.2 | 1.0 | 1.5 |
| | | D385 | 27 | 15768.2 | 11999.3 | 20720.9 | 27 | 1.1 | 0.9 | 1.3 |
| 3 | A/H9N2 AS03 | Pre | 30 | 12799.7 | 10297.3 | 15910.2 | - | - | - | - |
| | | D21 | 30 | 25189.3 | 20983.6 | 30238.0 | 30 | 2.0 | 1.6 | 2.4 |
| | | D42 | 30 | 31665.4 | 27405.9 | 36587.0 | 30 | 2.5 | 2.1 | 3.0 |
| | | D182 | 28 | 20570.2 | 16717.6 | 25310.5 | 28 | 1.6 | 1.4 | 1.9 |
| | A/H9N2 non-adjuvanted | Pre | 30 | 10888.1 | 8004.1 | 14811.3 | - | - | - | - |

| | | | | | | | | | | |
|---|---|---|---|---|---|---|---|---|---|---|
| | | D21 | 30 | 16592.8 | 13332.5 | 20650.4 | 30 | 1.5 | 1.3 | 1.8 |
| | | D42 | 30 | 19681.1 | 16293.6 | 23772.7 | 30 | 1.8 | 1.5 | 2.1 |
| | | D182 | 30 | 15471.1 | 11913.9 | 20090.4 | 30 | 1.4 | 1.2 | 1.6 |
| 4 | IIV4 non-adjuvanted | Pre | 30 | 11215.8 | 8015.8 | 15693.3 | - | - | - | - |
| | | D21 | 30 | 30437.5 | 24261.2 | 38186.2 | 30 | 2.7 | 2.0 | 3.7 |
| 5 | A/H5N1 AS03 Indonesia > Turkey | Pre | 26 | 8946.2 | 6147.1 | 13019.9 | - | - | - | - |
| | | D42 | 26 | 35325.3 | 25353.6 | 49218.9 | 26 | 3.9 | 2.8 | 5.6 |
| | | D182 | 26 | 22057.9 | 15456.7 | 31478.3 | 26 | 2.5 | 2.0 | 3.1 |
| | | D224 | 26 | 18748.2 | 13219.8 | 26588.7 | 26 | 2.1 | 1.7 | 2.6 |
| | | D549 | 25 | 21272.5 | 16188.8 | 27952.7 | 25 | 2.3 | 1.7 | 3.1 |
| | | D591 | 24 | 29023.7 | 22329.1 | 37725.4 | 24 | 3.1 | 2.2 | 4.2 |
| | | D729 | 24 | 22445.9 | 17120.0 | 29428.7 | 24 | 2.4 | 1.8 | 3.2 |
| | A/H5N1 AS03 Turkey > Turkey | Pre | 29 | 9205.1 | 6434.5 | 13168.6 | - | - | - | - |
| | | D42 | 28 | 10171.2 | 7035.3 | 14705.0 | - | - | - | - |
| | | D182 | 29 | 10123.1 | 6701.6 | 15291.4 | - | - | - | - |
| | | D224 | 28 | 43267.9 | 31807.8 | 58856.9 | 28 | 4.0 | 2.5 | 6.4 |
| | | D549 | 21 | 23507.6 | 16503.6 | 33484.1 | 21 | 2.0 | 1.5 | 2.8 |
| | | D591 | 20 | 33113.7 | 23879.1 | 45919.6 | 20 | 2.9 | 1.9 | 4.3 |
| | | D729 | 20 | 21894.3 | 15168.5 | 31602.4 | 20 | 1.9 | 1.3 | 2.7 |
| 6 | A/H5N1 AS03 Vietnam > Vietnam | Pre | 53 | 9340.1 | 7411.6 | 11770.5 | - | - | - | - |
| | | D21 | 53 | 35805.4 | 29821.1 | 42990.6 | 53 | 3.8 | 3.0 | 5.0 |
| | | D182 | 51 | 16123.6 | 13232.7 | 19646.1 | 51 | 1.7 | 1.5 | 2.1 |
| | | D365 | 50 | 14548.7 | 11989.4 | 17654.2 | 50 | 1.6 | 1.4 | 1.8 |
| | | D385 | 48 | 32675.5 | 27585.4 | 38704.8 | 48 | 3.6 | 2.8 | 4.8 |
| | | D549 | 49 | 21154.6 | 17597.8 | 25430.2 | 49 | 2.3 | 1.8 | 2.9 |
| | A/H5N1 AS03 Vietnam > Indonesia | Pre | 49 | 8409.3 | 6746.1 | 10482.4 | - | - | - | - |
| | | D21 | 49 | 40245.6 | 31912.9 | 50753.9 | 49 | 4.8 | 3.7 | 6.2 |
| | | D182 | 49 | 17337.3 | 13536.2 | 22206.0 | 49 | 2.1 | 1.7 | 2.5 |
| | | D365 | 48 | 16242.1 | 12854.0 | 20523.3 | 48 | 1.9 | 1.6 | 2.4 |
| | | D385 | 47 | 37096.0 | 29342.0 | 46899.1 | 47 | 4.5 | 3.4 | 6.0 |
| | | D549 | 45 | 19508.2 | 15715.0 | 24217.0 | 45 | 2.5 | 1.9 | 3.1 |

| | | | | | | | | | | |
|---|---|---|---|---|---|---|---|---|---|---|
| 7 | A/H5N1 AS03 | Pre | 32 | 2075.4 | 1458.3 | 2953.6 | - | - | - | - |
| | | D21 | 33 | 16552.4 | 9239.4 | 29653.7 | 32 | 8.8 | 5.0 | 15.6 |
| | | D42 | 33 | 22195.2 | 14601.3 | 33738.6 | 32 | 11.3 | 7.3 | 17.7 |
| | | D385 | 24 | 8661.1 | 5352.5 | 14014.8 | 23 | 4.1 | 2.3 | 7.2 |
| | Placebo | Pre | 17 | 2123.2 | 1042.1 | 4325.7 | - | - | - | - |
| | | D21 | 18 | 2203.3 | 1237.8 | 3922.1 | 17 | 1.1 | 0.9 | 1.2 |
| | | D42 | 18 | 2272.1 | 1310.1 | 3940.6 | 16 | 1.0 | 0.8 | 1.2 |
| | | D385 | 19 | 2394.7 | 1446.6 | 3964.0 | 16 | 1.4 | 1.0 | 2.2 |

Indonesia: A/Indonesia/5/05; Turkey: A/turkey/Turkey/1/2005; Vietnam: A/Vietnam/1194/2004

CI: confidence interval; D: day of measurement; ELISA: enzyme-linked immunosorbent assay; GMT: geometric mean titer; IIV4: inactivated quadrivalent influenza vaccine; LL: lower limit; MGI: mean geometric increase; UL: upper limit

**Supplementary Table 4. GMTs for anti-H2 full-length antibodies measured by ELISA following vaccination with adjuvanted pandemic vaccines and seasonal vaccine**

| Trial | Study group | Time point | GMT (95% CI) | | | | MGI [post-vaccination/pre-vaccination] (95% CI) | | | |
|---|---|---|---|---|---|---|---|---|---|---|
| | | | N | Point estimate | LL | UL | N | Point estimate | LL | UL |
| 1 | A/H1N1 AS03 | Pre | 27 | 3697.2 | 2695.5 | 5071.1 | - | - | - | - |
| | | D42 | 27 | 20783.2 | 16291.5 | 26513.4 | 25 | 6.0 | 4.4 | 8.0 |
| | | D182 | 29 | 12674.3 | 9909.2 | 16210.9 | 27 | 3.5 | 2.8 | 4.4 |
| 2 | A/H5N1 AS03 | Pre | 29 | 8366.5 | 5770.6 | 12130.2 | - | - | - | - |
| | | D42 | 29 | 51118.1 | 39968.6 | 65378.0 | 29 | 6.1 | 4.3 | 8.7 |
| | | D385 | 29 | 19903.0 | 15283.4 | 25919.0 | 29 | 2.4 | 1.9 | 3.0 |
| 3 | A/H9N2 AS03 | Pre | 30 | 14704.5 | 10160.7 | 21280.2 | - | - | - | - |
| | | D42 | 30 | 36716.0 | 27343.3 | 49301.5 | 30 | 2.5 | 2.0 | 3.1 |
| | | D182 | 28 | 23927.1 | 17505.5 | 32704.2 | 28 | 1.8 | 1.5 | 2.1 |
| 4 | IIV4 non-adjuvanted | Pre | 30 | 6435.6 | 4852.3 | 8535.5 | - | - | - | - |
| | | D21 | 30 | 12074.6 | 9400.2 | 15509.9 | 30 | 1.9 | 1.6 | 2.2 |
| 5 | A/H5N1 AS03 Indonesia > Turkey | Pre | 26 | 5832.4 | 4271.3 | 7964.0 | - | - | - | - |
| | | D42 | 26 | 23187.8 | 16597.1 | 32395.7 | 26 | 4.0 | 2.8 | 5.7 |
| | | D182 | 26 | 15364.4 | 11057.9 | 21347.9 | 26 | 2.6 | 2.1 | 3.3 |
| | | D549 | 25 | 12974.0 | 10089.8 | 16682.7 | 25 | 2.2 | 1.6 | 2.9 |
| | | D591 | 24 | 21467.9 | 16930.0 | 27222.1 | 24 | 3.6 | 2.7 | 4.6 |
| | | D729 | 24 | 14534.4 | 11280.4 | 18727.3 | 24 | 2.4 | 1.8 | 3.2 |
| | A/H5N1 AS03 Turkey > Turkey | D182 | 29 | 6914.7 | 4722.5 | 10124.4 | - | - | - | - |
| | | D224 | 28 | 34071.6 | 25325.9 | 45837.3 | 28 | 4.7 | 3.0 | 7.3 |
| | | D549 | 21 | 15142.3 | 10406.0 | 22034.2 | 21 | 2.0 | 1.5 | 2.6 |
| | | D591 | 20 | 24791.3 | 18324.3 | 33540.7 | 20 | 3.3 | 2.2 | 4.8 |
| | | D729 | 20 | 13865.1 | 9476.2 | 20286.9 | 20 | 1.8 | 1.3 | 2.5 |
| 6 | A/H5N1 AS03 Vietnam > Vietnam | Pre | 53 | 8735.3 | 6375.6 | 11968.5 | - | - | - | - |

| | | | | | | | | | | |
|---|---|---|---|---|---|---|---|---|---|---|
| | | D21 | 53 | 34355.8 | 26840.9 | 43974.8 | 53 | 3.9 | 3.0 | 5.1 |
| | | D365 | 50 | 14575.6 | 11067.3 | 19196.0 | 50 | 1.7 | 1.4 | 2.0 |
| | | D385 | 48 | 48534.4 | 39207.4 | 60080.4 | 48 | 5.6 | 4.0 | 8.0 |
| | | D549 | 49 | 23376.4 | 18227.7 | 29979.3 | 49 | 2.6 | 1.9 | 3.6 |
| | A/H5N1 AS03 Vietnam > Indonesia | Pre | 49 | 8633.4 | 6344.7 | 11747.7 | - | - | - | - |
| | | D21 | 49 | 40833.0 | 31899.1 | 52269.0 | 49 | 4.7 | 3.7 | 6.1 |
| | | D365 | 48 | 15958.9 | 12427.1 | 20494.4 | 48 | 1.8 | 1.5 | 2.3 |
| | | D385 | 47 | 51228.3 | 40614.9 | 64615.3 | 47 | 6.1 | 4.5 | 8.1 |
| | | D549 | 45 | 20685.0 | 16219.1 | 26380.5 | 45 | 2.5 | 2.0 | 3.2 |
| 7 | A/H5N1 AS03 | Pre | 29 | 1309.0 | 994.8 | 1722.5 | - | - | - | - |
| | | D42 | 33 | 20542.9 | 15121.2 | 27908.4 | 29 | 17.1 | 11.7 | 25.1 |
| | | D385 | 24 | 7258.3 | 4703.9 | 11199.9 | 22 | 5.5 | 3.1 | 9.8 |

Indonesia: A/Indonesia/5/05; Turkey: A/turkey/Turkey/1/2005; Vietnam: A/Vietnam/1194/2004

CI: confidence interval; D: day of measurement; ELISA: enzyme-linked immunosorbent assay; GMT: geometric mean titer; IIV4: inactivated quadrivalent influenza vaccine; LL: lower limit; MGI: mean geometric increase; UL: upper limit

**Supplementary Table 5. GMTs and MGIs for anti-H18 full-length antibodies measured by ELISA following vaccination with adjuvanted pandemic vaccines and seasonal vaccine**

| Trial | Study group | Time point | GMT (95% CI) | | | | MGI [post-vaccination/pre-vaccination] (95% CI) | | | |
|---|---|---|---|---|---|---|---|---|---|---|
| | | | N | Point estimate | LL | UL | N | Point estimate | LL | UL |
| 1 | A/H1N1 AS03 | Pre | 27 | 3261.8 | 2238.3 | 4753.4 | - | - | - | - |
| | | D42 | 27 | 15959.9 | 12897.9 | 19748.9 | 25 | 5.3 | 3.6 | 8.0 |
| | | D182 | 29 | 7791.5 | 6301.3 | 9634.2 | 27 | 2.5 | 1.8 | 3.4 |
| 2 | A/H5N1 AS03 | Pre | 29 | 5795.0 | 4011.8 | 8370.9 | - | - | - | - |
| | | D42 | 29 | 28492.2 | 21814.5 | 37214.0 | 29 | 4.9 | 3.5 | 6.9 |
| | | D385 | 29 | 11491.9 | 8848.8 | 14924.5 | 29 | 2.0 | 1.6 | 2.5 |
| 3 | A/H9N2 AS03 | Pre | 30 | 6893.6 | 5349.5 | 8883.5 | - | - | - | - |
| | | D42 | 30 | 19636.8 | 16519.2 | 23342.8 | 30 | 2.8 | 2.3 | 3.5 |
| | | D182 | 28 | 9859.5 | 7806.5 | 12452.5 | 28 | 1.4 | 1.2 | 1.8 |
| 4 | IIV4 non-adjuvanted | Pre | 30 | 5950.9 | 4212.2 | 8407.4 | - | - | - | - |
| | | D21 | 30 | 11025.1 | 8572.8 | 14178.9 | 30 | 1.9 | 1.4 | 2.4 |
| 5 | A/H5N1 AS03 Indonesia > Turkey | Pre | 26 | 4858.6 | 3288.8 | 7177.6 | - | - | - | - |
| | | D42 | 26 | 21291.6 | 15138.2 | 29946.1 | 26 | 4.4 | 3.1 | 6.3 |
| | | D182 | 26 | 10859.0 | 7483.4 | 15757.2 | 26 | 2.2 | 1.7 | 2.9 |
| | | D549 | 25 | 10935.8 | 8226.0 | 14538.4 | 25 | 2.2 | 1.6 | 2.9 |
| | | D591 | 24 | 15362.8 | 11801.3 | 19999.2 | 24 | 3.0 | 2.1 | 4.2 |
| | | D729 | 24 | 11917.5 | 8775.7 | 16184.0 | 24 | 2.3 | 1.7 | 3.2 |
| | A/H5N1 AS03 Turkey > Turkey | D182 | 29 | 4268.3 | 2736.3 | 6658.1 | - | - | - | - |
| | | D224 | 28 | 20162.7 | 14733.6 | 27592.3 | 28 | 4.5 | 2.7 | 7.3 |
| | | D549 | 21 | 12308.9 | 8353.2 | 18138.0 | 21 | 2.5 | 1.7 | 3.5 |
| | | D591 | 20 | 14064.2 | 9991.3 | 19797.4 | 20 | 2.8 | 1.9 | 4.2 |
| | | D729 | 20 | 10921.9 | 7313.7 | 16310.4 | 20 | 2.2 | 1.6 | 3.1 |
| 6 | A/H5N1 AS03 Vietnam > Vietnam | Pre | 53 | 5105.9 | 3937.3 | 6621.4 | - | - | - | - |

| | | | | | | | | | | |
|---|---|---|---|---|---|---|---|---|---|---|
| | | D21 | 53 | 22141.6 | 18114.4 | 27064.1 | 53 | 4.3 | 3.2 | 5.8 |
| | | D365 | 50 | 6743.4 | 5489.0 | 8284.6 | 50 | 1.4 | 1.2 | 1.6 |
| | | D385 | 48 | 14532.2 | 12132.7 | 17406.2 | 48 | 3.0 | 2.3 | 4.1 |
| | | D549 | 49 | 9467.3 | 7750.0 | 11565.2 | 49 | 1.9 | 1.5 | 2.5 |
| | A/H5N1 AS03 Vietnam > Indonesia | Pre | 49 | 4856.6 | 3949.9 | 5971.6 | - | - | - | - |
| | | D21 | 49 | 21054.9 | 16966.2 | 26129.0 | 49 | 4.3 | 3.4 | 5.6 |
| | | D365 | 48 | 7249.2 | 5785.2 | 9083.6 | 48 | 1.5 | 1.2 | 1.8 |
| | | D385 | 47 | 16655.6 | 13122.9 | 21139.2 | 47 | 3.5 | 2.7 | 4.5 |
| | | D549 | 45 | 10315.9 | 8230.5 | 12929.6 | 45 | 2.3 | 1.8 | 2.9 |
| 7 | A/H5N1 AS03 | Pre | 33 | 767.7 | 521.7 | 1129.8 | - | - | - | - |
| | | D42 | 33 | 12214.4 | 8399.6 | 17761.9 | 33 | 15.9 | 9.9 | 25.6 |
| | | D385 | 24 | 3974.2 | 2548.8 | 6196.8 | 24 | 4.7 | 2.7 | 8.4 |

Indonesia: A/Indonesia/5/05; Turkey: A/turkey/Turkey/1/2005; Vietnam: A/Vietnam/1194/2004

CI: confidence interval; D: day of measurement; ELISA: enzyme-linked immunosorbent assay; GMT: geometric mean titer; IIV4: inactivated quadrivalent influenza vaccine; LL: lower limit; MGI: mean geometric increase; UL: upper limit

## Supplementary Table 6. Clinical trial information

| Trial | GSK study name | GSK e-Track number | NCT identifier | Publication reference |
|---|---|---|---|---|
| 1 | Q-PAN H1N1-019 | 113536<br>https://www.gsk-studyregister.com/study/3953 | NCT00985673<br>https://www.clinicaltrials.gov/ct2/show/NCT00985673 | Langley *et al* 2012<br>https://www.ncbi.nlm.nih.gov/pubmed/23110320 |
| 2 | CC-PAN H5N1-001 | 114371<br>https://www.gsk-studyregister.com/study/4202 | NCT01236040<br>https://www.clinicaltrials.gov/ct2/show/NCT01236040 | Schuind *et al* 2015<br>https://www.ncbi.nlm.nih.gov/pubmed/25722291 |
| 3 | Q-PAN H9N2-001 | 116358<br>https://www.gsk-studyregister.com/study/4713 | NCT01659086<br>https://www.clinicaltrials.gov/ct2/show/NCT01659086 | Madan *et al* 2017<br>https://www.ncbi.nlm.nih.gov/pubmed/ 28720281 |
| 4 | FLU D-QIV-015 | 201251<br>https://www.gsk-studyregister.com/study/5327 | NCT02207413<br>https://www.clinicaltrials.gov/ct2/show/NCT02207413 | Claeys *et al* 2018<br>https://www.ncbi.nlm.nih.gov/pubmed/ 29669531 |
| 5 | Q-PAN-005 | 110624<br>https://www.gsk-studyregister.com/study/3305 | NCT00719043<br>https://www.clinicaltrials.gov/ct2/show/NCT00719043 | Langley *et al* 2015<br>https://www.ncbi.nlm.nih.gov/pubmed/ 25448092 |
| 6 | H5N1-012 | 107495<br>https://www.gsk-studyregister.com/study/3126 | NCT00430521<br>https://www.clinicaltrials.gov/ct2/show/NCT00430521 | Schwarz *et al* 2009<br>https://www.ncbi.nlm.nih.gov/pubmed/ 19856521<br>Gillard *et al* 2013<br>https://www.ncbi.nlm.nih.gov/pubmed/22405557 |
| 7 | Q-PAN H5N1-AS03-021 | 114464<br>https://www.gsk-studyregister.com/study/4233 | NCT01310413<br>https://www.clinicaltrials.gov/ct2/show/NCT01310413 | Kosalaraksa *et al* 2015<br>https://www.ncbi.nlm.nih.gov/pubmed/ 25293368 |

**Supplementary Figure 1. GMTs and MGIs for anti-H18 full-length antibodies measured by ELISA following vaccination with adjuvanted pandemic vaccines and seasonal vaccine**

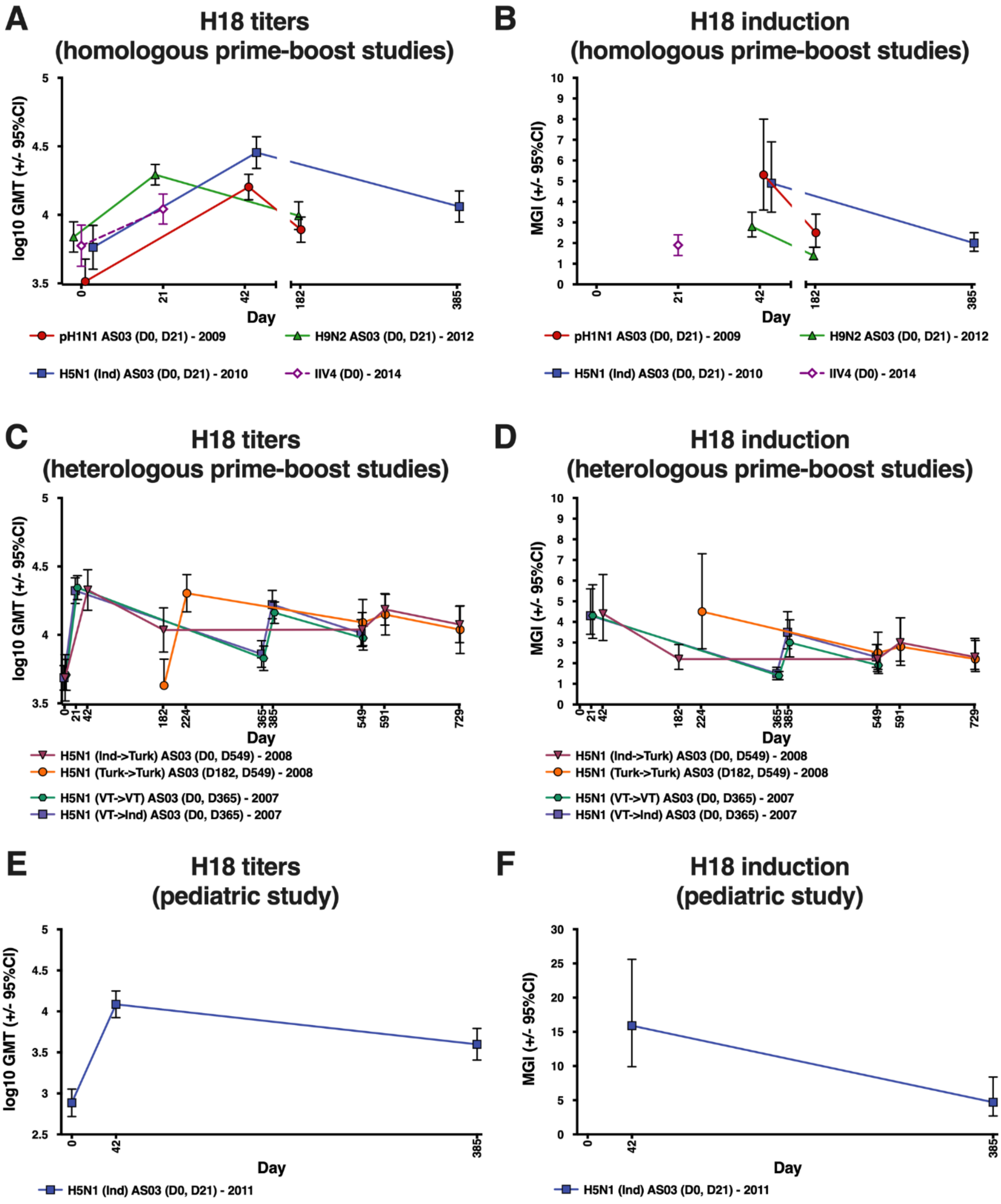


Ind: A/Indonesia/5/05; Turk: A/turkey/Turkey/1/2005; VT: A/Vietnam/1194/2004

Error bars indicate 95% confidence intervals.

ELISA: enzyme-linked immunosorbent assay; GMT: geometric mean titer; IIV4: inactivated quadrivalent influenza vaccine; MGI; mean geometric increase

**Supplementary Figure 2. GMTs and MGIs for vaccine-heterosubtypic antibodies (anti-A/H5N8) measured by microneutralization assay following vaccination with adjuvanted pandemic vaccines and seasonal vaccine**

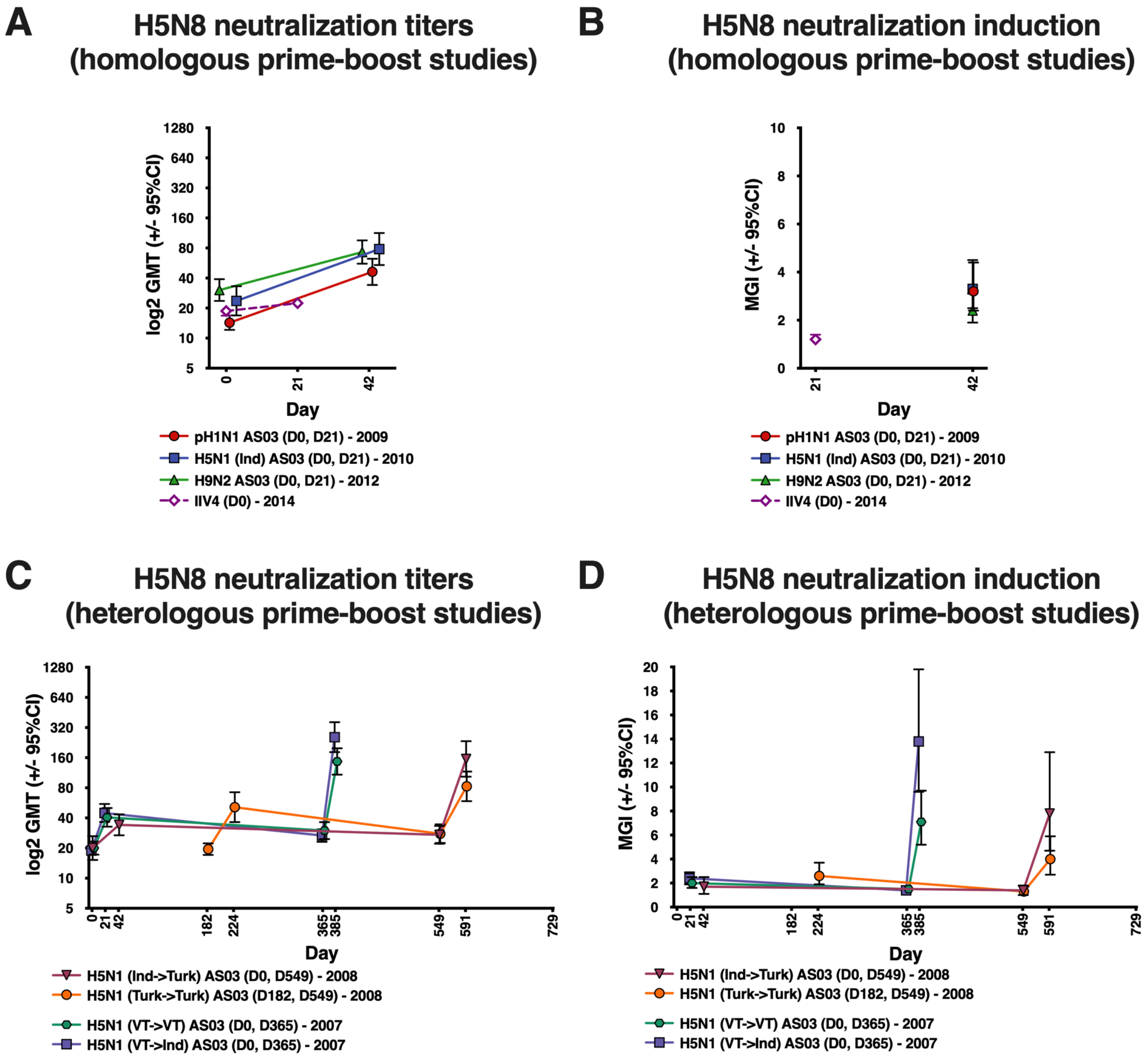


Error bars indicate 95% confidence intervals.

Ind: A/Indonesia/5/05; Turk: A/turkey/Turkey/1/2005; VT: A/Vietnam/1194/2004

GMT: geometric mean titer; IIV4: inactivated quadrivalent influenza vaccine; MGI; mean geometric increase

**Supplementary Figure 3. GMTs and MGIs for vaccine-heterosubtypic antibodies (anti avian-like swine A/H1N1) measured by microneutralization assay following vaccination with adjuvanted pandemic vaccines and seasonal vaccine**

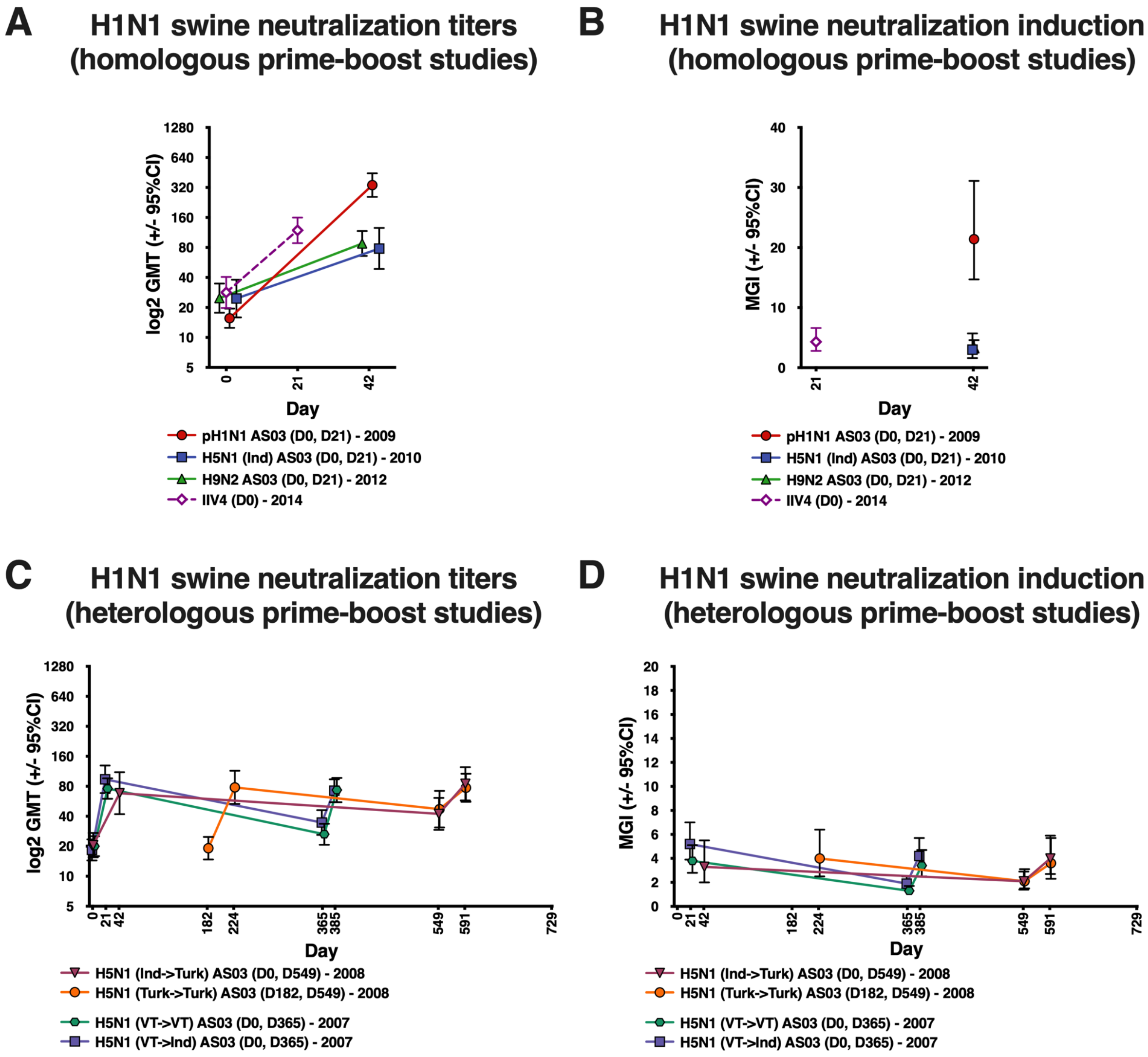


Error bars indicate 95% confidence intervals.

Ind: A/Indonesia/5/05; Turk: A/turkey/Turkey/1/2005; VT: A/Vietnam/1194/2004

GMT: geometric mean titer; IIV4: inactivated quadrivalent influenza vaccine; MGI; mean geometric increase

**Supplementary Figure 4. GMTs and MGIs for vaccine-heterosubtypic antibodies (anti-A/H1N1pdm09) measured by microneutralization assay following vaccination with adjuvanted pandemic vaccines and seasonal vaccine**

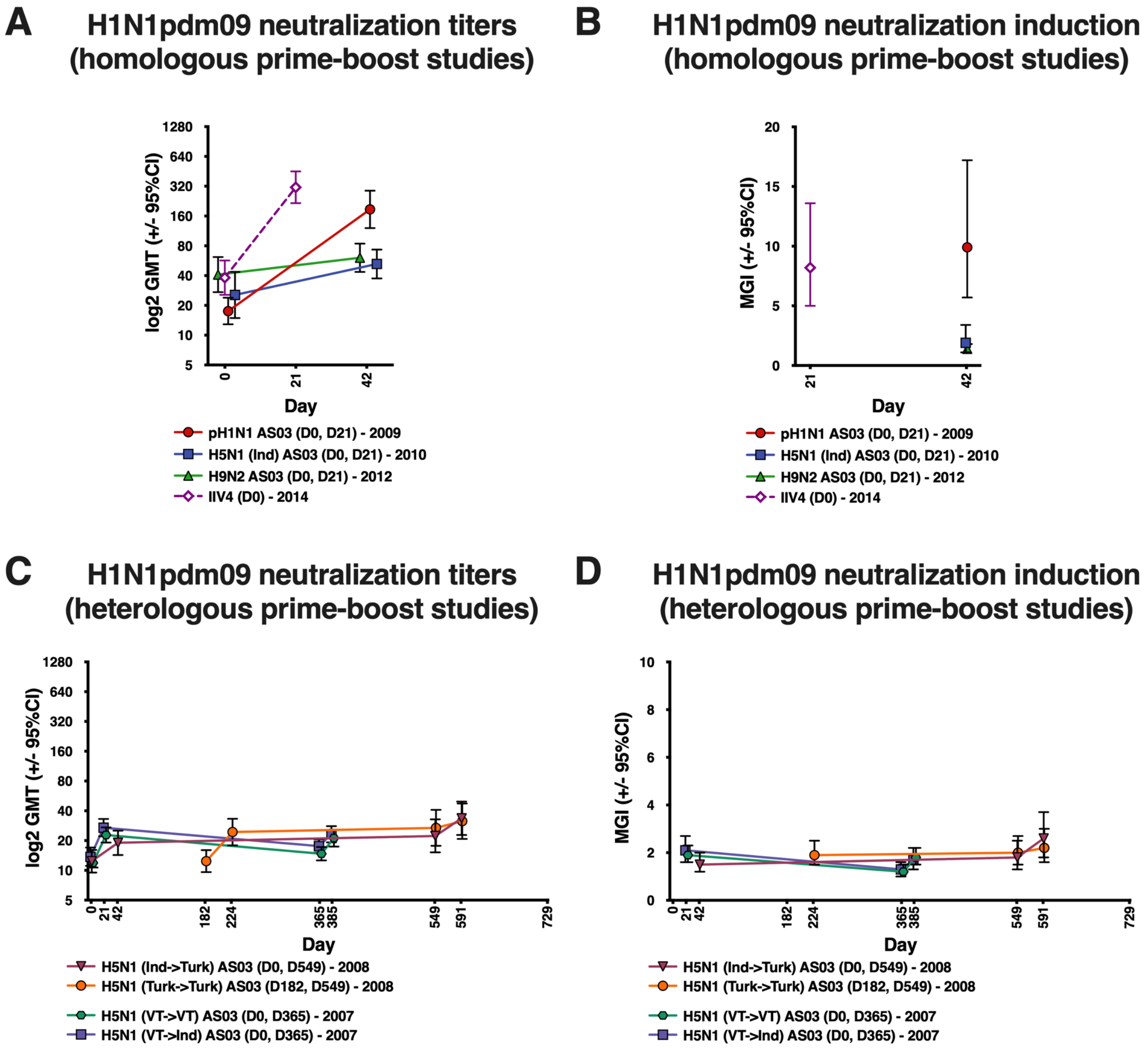


Error bars indicate 95% confidence intervals.

Ind: A/Indonesia/5/05; Turk: A/turkey/Turkey/1/2005; VT: A/Vietnam/1194/2004

GMT: geometric mean titer; IIV4: inactivated quadrivalent influenza vaccine; MGI; mean geometric increase

**Supplementary Figure 5. Area under the curve for anti-H1 stalk antibodies measured by ELISA following vaccination of mice with adjuvanted and non-adjuvanted pandemic vaccines and seasonal vaccine (separate study)**

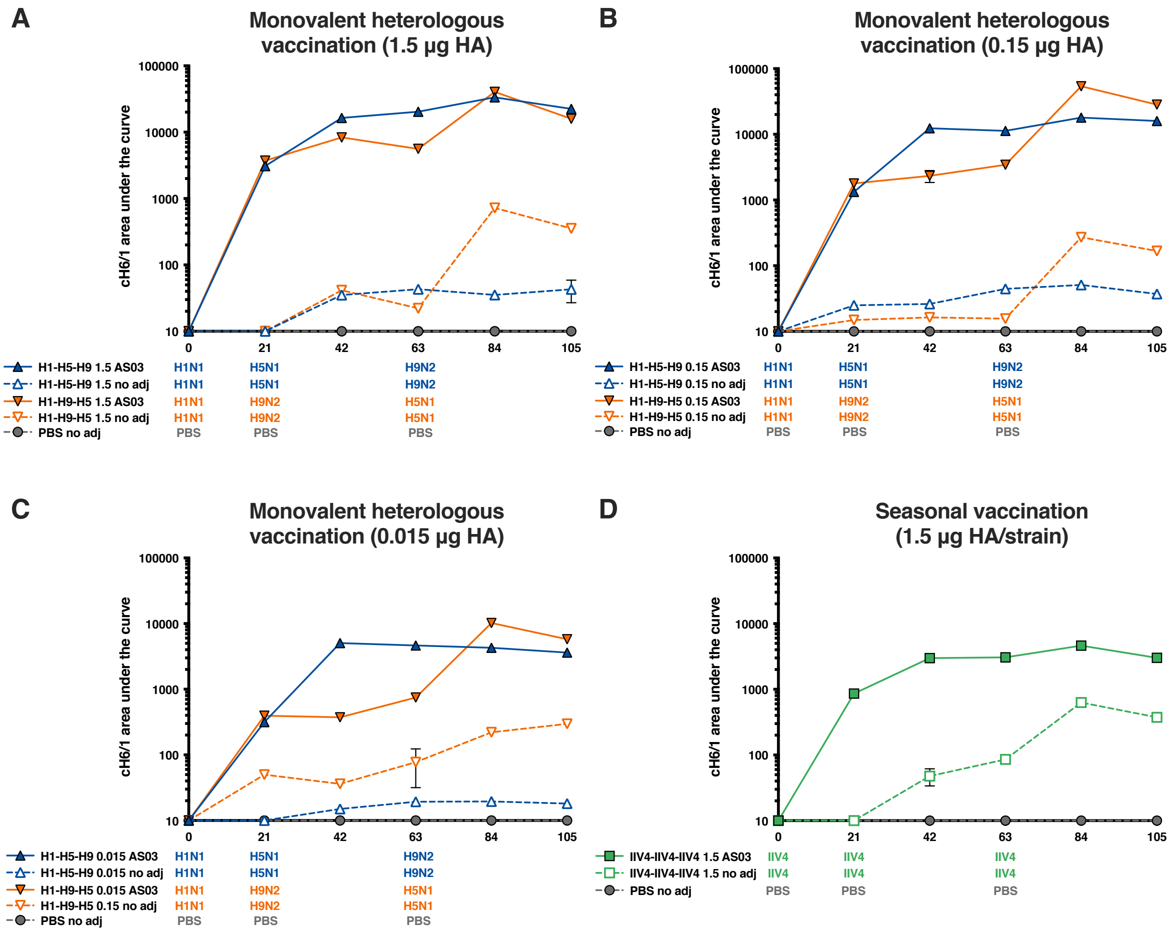


Female 6-8-week-old C57BL/6 mice (n=14-15 mice/group) were vaccinated with either (1) divergent monovalent split virus vaccines (A/H1N1, A/H5N1, A/H9N2) containing full-length HAs with naturally occurring exotic heads on a GrA1 stalk or (2) seasonal inactivated quadrivalent vaccine (IIV4). All vaccines were formulated with or without AS03 adjuvant (0.593 mg α- tocopherol per vaccine dose) and were manufactured by GSK Vaccines, Ste-Foy, Quebec City, Canada.

The monovalent split virus vaccines were given at a dose of either 1.5 **(A)**, 0.15 **(B)**, or 0.015 µg **(C)** of HA. A/H1N1 vaccine was administered on day 0, followed by either (1) A/H5N1 on day 21 and A/H9N2 on day 63 or (2) A/H9N2 on day 21 and A/H5N1 on day 63. IIV4 was given at 1.5 µg of HA per strain **(D)** on days 0, 21, and 63. Control mice were vaccinated with phosphate buffered saline (PBS). Mice were injected with a total volume of 50 µL per immunization.

Sera were collected on days 0, 21, 42, 63, 84 and 105, pooled, tested in triplicate by ELISA for H1 stalk-specific antibodies (using a chimeric 6/1 construct). Area under the curve (AUC) values were

calculated as previously described.[1] All mouse procedures were approved in advance by the Institutional Animal Care Committee at the Institut Armand Frappier (Laval, Quebec City, Canada) according to the guidelines of the Canadian Council on Animal Care.

Symbols show mean values and error bars show the standard error of the mean. AUC values of 10 were set as the assay baseline.

AS03-adjuvanted vaccines (solid lines) elicited higher levels of HA stalk antibodies compared with non-adjuvanted vaccines (dashed lines). At 1.5 µg, the AS03-adjuvanted monovalent split virus vaccines elicited higher HA stalk antibody titers compared with mice vaccinated with IIV4 **(A, D)**, similar to previous studies.[2] An interesting difference between A/H5N1 and A/H9N2 vaccines was observed **(A-C)**. A/H9N2 vaccination did not effectively boost HA stalk antibody levels compared with A/H5N1 vaccination on either day 21 or 63 (orange or blue lines, respectively). However, mice that received a final A/H5N1 vaccination on day 63 (orange lines) elicited higher HA stalk antibody levels on days 84 and 105, indicating that A/H9N2 vaccination provided some priming effect.

**Supplementary References**

1. Choi A, Bouzya B, Cortés Franco KD, et al. Chimeric hemagglutinin-based influenza virus vaccines induce protective stalk-specific humoral immunity and cellular responses in mice. Immunohorizons 2019; 3:133−148.

2. Nachbagauer R, Kinzler D, Choi A, et al. A chimeric haemagglutinin-based influenza split virion vaccine adjuvanted with AS03 induces protective stalk-reactive antibodies in mice. NPJ Vaccines 2016; 1:16015.
